# Novel taxa of Acidobacteriota implicated in seafloor sulfur cycling

**DOI:** 10.1038/s41396-021-00992-0

**Published:** 2021-05-12

**Authors:** Mathias Flieder, Joy Buongiorno, Craig W. Herbold, Bela Hausmann, Thomas Rattei, Karen G. Lloyd, Alexander Loy, Kenneth Wasmund

**Affiliations:** 1 Division of Microbial Ecology, Centre for Microbiology and Environmental Systems Science, University of Vienna, Vienna, Austria; 2 Department of Microbiology, University of Tennessee, Knoxville, TN USA; 3 Joint Microbiome Facility of the Medical University of Vienna and the University of Vienna, Vienna, Austria; 4 Department of Laboratory Medicine, Medical University of Vienna, Vienna, Austria; 5 Division of Computational Systems Biology, Centre for Microbiology and Environmental Systems Science, University of Vienna, Vienna, Austria; 6 Austrian Polar Research Institute, Vienna, Austria; 7 Center for Microbial Communities, Department of Chemistry and Bioscience, Aalborg University, Aalborg, Denmark; 8 Present Address: Division of Natural Sciences, Maryville College, Maryville, TN USA

**Keywords:** Environmental microbiology, Environmental sciences

## Abstract

Acidobacteriota are widespread and often abundant in marine sediments, yet their metabolic and ecological properties are poorly understood. Here, we examined metabolisms and distributions of Acidobacteriota in marine sediments of Svalbard by functional predictions from metagenome-assembled genomes (MAGs), amplicon sequencing of 16S rRNA and dissimilatory sulfite reductase (*dsrB*) genes and transcripts, and gene expression analyses of tetrathionate-amended microcosms. Acidobacteriota were the second most abundant *dsrB*-harboring (averaging 13%) phylum after Desulfobacterota in Svalbard sediments, and represented 4% of *dsrB* transcripts on average. Meta-analysis of *dsrAB* datasets also showed Acidobacteriota *dsrAB* sequences are prominent in marine sediments worldwide, averaging 15% of all sequences analysed, and represent most of the previously unclassified *dsrAB* in marine sediments. We propose two new Acidobacteriota genera, *Candidatus* Sulfomarinibacter (class Thermoanaerobaculia, “subdivision 23”) and *Ca*. Polarisedimenticola (“subdivision 22”), with distinct genetic properties that may explain their distributions in biogeochemically distinct sediments. *Ca*. Sulfomarinibacter encode flexible respiratory routes, with potential for oxygen, nitrous oxide, metal-oxide, tetrathionate, sulfur and sulfite/sulfate respiration, and possibly sulfur disproportionation. Potential nutrients and energy include cellulose, proteins, cyanophycin, hydrogen, and acetate. A *Ca*. Polarisedimenticola MAG encodes various enzymes to degrade proteins, and to reduce oxygen, nitrate, sulfur/polysulfide and metal-oxides. 16S rRNA gene and transcript profiling of Svalbard sediments showed *Ca*. Sulfomarinibacter members were relatively abundant and transcriptionally active in sulfidic fjord sediments, while *Ca*. Polarisedimenticola members were more relatively abundant in metal-rich fjord sediments. Overall, we reveal various physiological features of uncultured marine Acidobacteriota that indicate fundamental roles in seafloor biogeochemical cycling.

## Introduction

Bacteria of the phylum Acidobacteriota (also known as “Acidobacteria”) are highly diverse and inhabit a vast array of environments on Earth, yet the properties of various Acidobacteriota lineages remain poorly understood [1–6]. Knowledge regarding the functions and ecology of Acidobacteriota is biased to isolates and genomes obtained from soils, where they are especially prevalent and often dominate microbial communities [3, 4]. Soil-derived Acidobacteriota are generally known as aerobic heterotrophs that utilize various carbohydrates including polysaccharides like chitin or cellulose [3, 7, 8]. Some Acidobacteriota known from other environments have unique physiological properties, such as the ability to reduce iron [9], perform phototrophy [9, 10], or exhibit thermophilic lifestyles [11]. Members of Acidobacteriota subdivisions 1 and 3 from peatland and permafrost soils have the potential to dissimilate inorganic and/or organic sulfur compounds [2, 12]. In comparison to terrestrial Acidobacteriota, very little is known about Acidobacteriota in marine systems.

Acidobacteriota 16S rRNA genes or genomes are frequently detected in marine environments including ocean waters, marine sponges, hydrothermal vents, or sediments [13–17]. Studies of 16S rRNA genes in marine sediments showed that Acidobacteriota are widespread and reach relative abundances in amplicon libraries of up to 23% [18–23]. This suggests they play important roles in microbial community functioning and biogeochemical processes, although our knowledge regarding their specific roles in sediments remains limited. A recent stable isotope probing study showed some Acidobacteriota in deep-sea sediments are capable of fixing nitrogen [24]. Acidobacteriota were also shown to be active under sulfidic conditions in ^18^O–H_2_O incubations with estuarine sediment, by stable isotope-labeling of their 16S rRNA and dissimilatory sulfite reductase (*dsrB*) genes [6]. One novel Acidobacteriota metagenome-assembled genome (MAG) (*Ca.* Guanabacteria) encoded genes for the CO dehydrogenase/CO-methylating acetyl-CoA synthase complex and heterodisulfide reductases, indicating a possible anaerobic lifestyle [25].

Marine sediments are a massive global habitat for microorganisms [26], with average cell densities of microorganisms up to 10^9^ cells per cm^3^ in surface sediments of organic-rich sediments [27]. Substantial amounts of organic matter are processed in marine sediments, which makes them a critical component of marine and global biogeochemical cycles [28]. Marine sediments are often stratified with respect to redox states, whereby oxygen is typically depleted within millimeters to centimetres below the surface at sites where organic inputs are relatively high [29]. Vast expanses of sediments are therefore anoxic, and many microorganisms survive via anaerobic lifestyles, such as fermentation, or respiration of nitrate, metals, sulfate or CO_2_. Sulfate is abundant in sediments and is used by sulfite/sulfate-reducing microorganisms (SRMs) as an electron acceptor for anaerobic respiration. Sulfate reduction is estimated to facilitate ~29% of organic matter degradation in marine sediments globally [26, 28]. The sulfur-cycle is therefore a major driver of microbial life and biogeochemical cycling in the seafloor, and therefore understanding the microorganisms that catalyze sulfur cycling is of great importance.

Because sulfate reduction is a major process in marine sediments, the activities, distributions, and diversity of SRMs have been relatively well studied [28, 30, 31]. Members of the Desulfobacterota (formerly “Deltaproteobacteria”) are known as abundant SRMs in marine sediments, playing key roles in anaerobic food webs by utilizing fermentation products released by primary degraders of organic matter [32–34]. They are also represented by various isolates, and many have been subject to genomic and physiological studies [35]. Surveys of functional marker genes for sulfite/sulfate reducers in marine sediments, i.e., of *dsrAB*, have repeatedly shown that *dsrAB* from the phylum Desulfobacterota are typically the dominant *dsrAB*-harboring group in marine sediments, but importantly, that several other lineages of uncultivated *dsrAB*-harboring organisms are also abundant and prevalent [36]. Recently, some *dsrAB* sequences in marine sediments have been inferred to belong to Acidobacteriota [6, 37], although nothing is known about the metabolic properties, genomes, or the sulfur-dissimilating pathways of the organisms that harbor these genes. Identifying and understanding these undescribed *dsrAB*-harboring microorganisms is therefore critical for understanding the microbial groups that drive sulfur cycling in marine sediments.

In this study, we aimed to gain insights into the metabolic potential of uncultured Acidobacteriota lineages in marine sediments, as well as their diversity and distributions. We therefore recovered metagenome-assembled genomes (MAGs) from abundant Acidobacteriota populations present in marine fjord sediments of Svalbard, and predicted their metabolic features. Focus was placed on MAGs from the class Thermoanaerobaculia of the Acidobacteriota, which represent a newly described lineage of *dsrAB*-harboring organisms that may be important sulfur cycling bacteria in marine sediments. These analyses were complemented with comparative genomics, incubation experiments, transcript analyses, and analyses of Acidobacteriota distributions in Svalbard sediments and publicly available datasets, together revealing they may play various roles in sedimentary biogeochemical cycles, and that they are a prominent group of sulfur-dissimilating organisms.

## Materials and methods

### Sample collection

Marine sediments were collected from Smeerenburgfjorden, Kongsfjorden and Van Keulenfjorden, of Svalbard, Norway, in July 2016 and/or June 2017 with the vessel “MS Farm”. Extensive biogeochemical data for these sites is available from previous studies [38–42]. Maps of sample locations are presented in Michaud et al. [41]. From Smeerenburgfjorden, individual core samples were taken from three stations: station GK (79°38.49N, 11°20.96E), station J (79°42.83N, 11°05.10E), and station GN (79°45.01N, 11°05.99E), with the station J cores being taken in both 2016 and 2017. Duplicate core samples from Van Keulenfjorden were taken from sites AC (77°32.260′N, 15°39.434′E) and AB (77°35.249′N, 15°05.121′E). A sample was also taken from Kongsfjorden station F (78°55.075′N, 12°15.929′E) [43]. For molecular biological analyses, samples were taken with HAPS [44] or Rumohr corers [45]. Details of core subsampling procedures are provided in the Supplementary Information.

### Microcosm incubations with tetrathionate additions

To examine tetrathionate reductase gene expression, a sediment incubation experiment was performed. A sediment slurry was prepared inside an anoxic glove box (nitrogen atmosphere containing 2% hydrogen and 10% CO_2_), from samples collected in 2017 from 5 to 10 cmbsf at Station J, Smeerenburgfjorden. Sediments had been stored at 4 °C for 6 months prior to the experiment. Anoxic artificial seawater [46] containing 28 mM sulfate was well-mixed with a 2:1 ratio with sediment. Autoclaved serum bottles (250 ml) that were left in the anoxic glove box overnight prior to the experiment to remove traces of oxygen, were filled with 30 ml of sediment slurry. All microcosms received a small amount of organic material to boost heterotrophic activity, i.e., yeast extract (0.22 mg ml^−1^) (Oxoid). All experiments were set up in triplicates. The experimental treatments included additions of: (i) tetrathionate (500 µM final), or (ii) “no substrate” controls. All microcosms were sealed with autoclaved butyl rubber stoppers. The experiment was incubated at 4 °C for 8 days, and samples were taken at the start of the experiments, day 1 and day 8. Additional tetrathionate (to make 500 µM additional) was spiked into the microcosms on day 5. Subsampling was done inside the anoxic glove box with microcosms placed on ice-pads to reduce warming of the samples. Samples (250 µl) were taken for DNA/RNA-based analyses, kept on the ice-pads in the anoxic glove box and transferred immediately to dry-ice outside the glove box, and stored at −80 °C.

### Nucleic acid extractions and reverse transcription

For amplicon-based analyses, DNA and RNA was extracted from the sediment core samples (~500 µl) and microcosm samples (*~*250 µl) using the RNeasy PowerSoil Total RNA Kit (Qiagen) according to the manufacturer’s instructions. Additionally, a phenol/chloroform based extraction method [47], was used to extract total nucleic acids from sediment samples from station J sampled in July 2016 (Supplementary Information). Eluted nucleic acids were stored in molecular biology grade water at −80 °C. Aliquots for DNA-based analyses were used as eluted, while aliquots for RNA-based analyses were DNase-treated using the TURBO DNA-free kit (Thermo Fisher), followed by reverse transcription of the RNA to cDNA using the RevertAid First Strand cDNA Synthesis Kit (Thermo Fisher) according to the manufacturer’s instructions. To test if any DNA remained in the RNA samples after the DNase digestion step, control samples were processed as above except the RevertAid M-MuLV Reverse Transcriptase was excluded. These controls were checked for DNA by PCR using 16S rRNA gene targeting primers (described below).

Sediment samples from 2016 were used for metagenome sequencing. DNA was extracted by the Vienna group from 3 to 5 mL of sediment from varying depths or microcosms derived from station J, Smeerenburgfjorden, and 18 centimeters below seafloor (cmbsf) from station AC of Van Keulenfjorden (Supplementary Table 1) using the DNeasy PowerSoil Kit (Qiagen) according to the manufacturer’s protocol. DNA was also extracted by the Knoxville group from 2 g of sample spanning 0–5 cmbsf from site AB of Van Keulenfjorden and site F of Kongsfjorden (Supplementary Table 1), using the RNeasy PowerSoil Kit (Qiagen) with DNA elution following the manufacturer’s protocol.

### Metagenome sequencing and genome binning

DNA libraries were prepared from individual samples (detailed in Supplementary Information) and sequenced using 2 × 150 bp paired-end mode with a HiSeq 3000 (Illumina) instrument at the Biomedical Sequencing Facility (BSF), Vienna. Metagenomic libraries were generated from the combined extracts from the first 5 cm (spanning 0–5 cm downcore) in sites AB and F in the Center for Environmental Biotechnology, Knoxville, using HiSeq (Illumina), 2 × 250 bp in paired-end mode [43]. Sequencing output summaries are provided in Supplementary Table 1.

Sequence reads were quality filtered, trimmed, and normalized as described in the Supplementary Information. Processed reads from each sample were assembled separately using IDBA-UD (version 1.1.1) [48] with default settings and the following options: --min_contig 500 --pre_correction. Reads from site F (Kongsfjorden) were assembled via metaSPAdes (version 3.11) [49] with kmer sizes set to 21, 33, 55, 77, 99, and 127 to find the best assembly. All other samples were assembled using metaSPAdes on the KBase server [50] with the default parameters and following options: minimum contig length of 1000 bp, and kmer sizes of 21, 33, and 55. All samples were also assembled using Megahit [51] on the KBase server using default parameters. KBase Narratives are available/searchable from user “jbuongio”.

Coverage profiles of assembled unbinned contigs were acquired by mapping trimmed reads (not normalized) to assemblies using BWA [52] and SAMtools [53]. Contigs from each assembly were then binned into MAGs using MetaBat2 (using each binning strategy) (version 2.12.1) [54], CONCOCT (version 0.4.1) [55], and MaxBin2 (version 2.2.4) [56]. MAG collections derived from each binning strategy, from all respective assemblies, were then aggregated using DasTool (version 1.1.0) (Supplementary Fig. 1A) [57]. Finally, all MAGs were dereplicated using dRep (version 1.4.3) [58], with the options: an average nucleotide identity (ANI) of 98% was used as cutoff to dereplicate MAGs from the secondary ANI comparison [59], and MAGs >50% complete and <10% contamination were retained. Estimations of completeness and degree of contamination of MAGs were obtained by CheckM (version 1.0.7) [60]. Read mapping to compare relative abundances of read recruitment to MAGs was performed using BBMap [61], with the default settings and “minid” of 0.99 for the minimum identity threshold. Taxonomic affiliations of MAGs were determined with GTDB-Tk [62]. ANI comparisons of MAGs were obtained using JSpeciesWS server based on BLASTN (“ANIb”) [63] and ANIcalculator [64].

### Gene annotations and in silico analyses of inferred proteins

Calling of genes and automatic annotations were obtained using the RAST server with default settings and the “classic” annotation pipeline [65]. Functions of predicted proteins of interest were manually checked after searches with BLASTP [66] against the NCBI-nr and SWISS-PROT databases [67] (>25% identity), and the Conserved Domain Database (CDD) [68] (default expect value of 0.01). Functions were together manually assessed by considering various features including: (i) proteins had >25% amino acid identity to biochemically characterized proteins present in SWISS-PROT databases, (ii) considering if functional domains were detected by CDD, (iii) if genes for proteins being examined were present in gene clusters with functionally related proteins (e.g., of same enzymatic complex and/or biochemical pathways [69, 70]), (iv) after being evaluated using literature searches and the MetaCyc database [71], and (v) in certain cases, phylogenetic comparisons between related protein sequences (described below). Methods for further annotations and protein sequence analyses and for gene content comparisons among MAGs are described in the Supplementary Information.

### MiSeq amplicon sequencing and sequence analyses

For amplification of bacterial and archaeal 16S rRNA genes or transcripts (cDNA) from Smeerenburgfjorden sediments, the primers 515F (5′-GTGYCAGCMGCCGCGGTAA-3′) [72] and 806R (5′-GGACTACNVGGGTWTCTAAT-3′) [73] including a 5′-head sequence for 2-step PCR barcoding [74], were used (further details in  Supplementary Information). Triplicate PCRs were performed in the first-step PCR, and then pooled before the second barcoding PCR. Slight variants of these PCR primers 515F and 806R [75] for 16S rRNA genes were used in amplicon sequencing profiling of sediments from Van Keulenfjorden in a previous study, although a standard “one-step PCR” approach was used [40]. Amplicon pools were extracted from the raw sequencing data using the FASTQ workflow in BaseSpace (Illumina) with default parameters. Demultiplexing was performed with the python package demultiplex (Laros JFJ, github.com/jfjlaros/demultiplex) allowing one mismatch for barcodes and two mismatches for linkers and primers. DADA2 [76] was used for demultiplexing amplicon sequencing variants using a previously described standard protocol [77]. FASTQ reads 1 and 2 were trimmed at 220 nt and 150 nt, respectively, with allowed expected errors of 2. Taxonomy was assigned to 16S rRNA gene/transcript sequences based on SILVA taxonomy (release 138) using the naïve Bayesian classification method as implemented in *mothur* [78]. Amplicon sequence datasets were analysed with the Rhea pipeline [79] implemented in R (https://www.r-project.org/).

Primers DSR-1762Fmix and DSR-2107Rmix, including a 5′-head sequence for barcoding, were used for amplification of *dsrB*-genes or -transcripts (cDNA) [80] (further details in Supplementary Information). Triplicate PCRs were performed in the first-step PCR, and then pooled before the second barcoding PCR. Raw reads were then processed as previously described [74, 80], into *dsrB* operational taxonomic units (OTUs) with >99% identity. Classification of amplicon-derived DsrB sequences was performed using a combined phylogenetic and naïve Bayesian classification approach as previously described [80].

Methods for analyses of publicly available 16S rRNA gene and *dsrAB* sequence datasets are described in the Supplementary Information.

### Quantitative reverse-transcription PCR

RT-qPCR assays targeting the octaheme cytochrome tetrathionate reductase (*otr*) and *dsrB* genes of MAG AM3-C were performed using the newly-designed primers TetraC-C-F (5′-CACCACGACCTGTCTCGG-3′) and TetraC-C-R (5′-CCCCCTGGAGTTCTTGGT-3′), and Acido-dsrB-F (5′-GGAGAACTATGGGAAGTGGG-3′) and Acido-dsrB-R (5′-GTTGAGGCAGCACGCGTA-3′). Primers 1329-B-F (5′-AACCTTTGGGCGATTTCTCG-3′) and 1329-B-R (5′-GAGAGAGTGGCAACGTGAAC-3′) targeting the DNA-directed RNA polymerase alpha subunit gene of MAG AM3-C were used to examine expression of a housekeeping gene. Details of RT-qPCR assay conditions are presented in the Supplementary Information. Relative-fold changes of target gene transcripts were calculated relative to the housekeeping gene of the alpha subunit of DNA-directed RNA polymerase by the 2^–ΔCt^ calculation as described by Schmittgen and Livak [81].

### Phylogenetic analyses

A phylogenomic maximum-likelihood tree was created using the IQ-TREE web-server with automatic substitution model selection and ultrafast bootstrapping (1000×) [82] using an alignment of concatenated protein sequences derived from single copy marker genes retrieved from CheckM [60]. The tree was visualized with iTol [83]. Phylogenetic analysis of 16S rRNA was performed in ARB [84] using the SILVA database release 138 [85], and *dsrAB* sequences were also analysed using ARB using previously described database [36, 80] (Supplementary Information). Phylogenetic analyses of all other protein sequences were performed using the IQ-TREE web-server with automatic substitution model selection and ultrafast bootstrapping (1000×) [82]. For the Complex-Iron-Sulfur-Molybdoenzyme (CISM) tree, query protein sequences were added to a previous alignment of CISM protein sequences [86], using MAFFT using the “add full length sequences” option (--add) [87]. All other protein sequence alignments were made de novo with MUSCLE [88] within Mega6 [89].

### Sequence and MAG accessions

Metagenomic sequence reads from Van Keulenfjorden and Kongsfjorden samples are available under NCBI-Genbank Bioproject PRJNA493859. Metagenomic sequence reads, and 16S rRNA gene and *dsrB* sequence reads from Smeerenburgfjorden samples are available under NCBI-Genbank Bioproject PRJNA623111. MAGs are available under NCBI-Genbank Bioproject PRJNA623111, with Biosample accessions SAMN15691661-SAMN15691666.

## Results

### Recovery of novel Acidobacteriota genomes from marine sediments

Metagenomic sequencing and genome binning was performed from DNA extracted and sequenced from sediments originating from three fjords from Svalbard, Norway (Supplementary Table 1). Our genome binning strategy based on multiple assemblies and multiple binning algorithms recovered more MAGs with higher completeness, as compared to applying multiple binning approaches based on single assembly approaches (Supplementary Fig. 1A, B). From the dereplicated MAGs (*n* = 97), four represented populations of the phylum Acidobacteriota and were chosen for in-depth analyses.

**Fig. 1:**
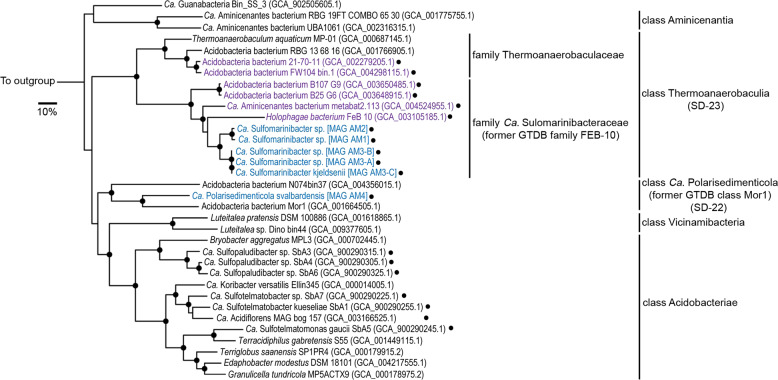
Phylogenomic analysis reveals novel Acidobacteriota taxa in marine sediments. Maximum-likelihood tree of concatenated protein sequences from MAGs and genomes. Single marker genes were retrieved with CheckM. Highlighted in blue are MAGs obtained in this study. Highlighted in purple are *dsrAB*-containing MAGs obtained from the NCBI database from the class Thermoanaerobaculia. The genus *Ca*. Acidiflorens is represented by the most complete MAG (GCA_003166525.1) from the corresponding study [12]. Our phylogenomic analysis showed that one MAG that was previously assigned to *Ca*. Aminicenantes (GCA_004524955.1), recovered from the Bothnian Sea [146], is affiliated with the newly proposed family *Ca*. Sulfomarinibacteraceae. Black dots indicate *dsrAB*-containing genomes/MAGs. Bootstrap values with >90% are indicated with filled black circles on nodes. *Nitrospina gracilis* 3/211 (GCA 000341545.2) was used as an outgroup. The scale bar represents 10% sequence divergence.

**Fig. 2:**
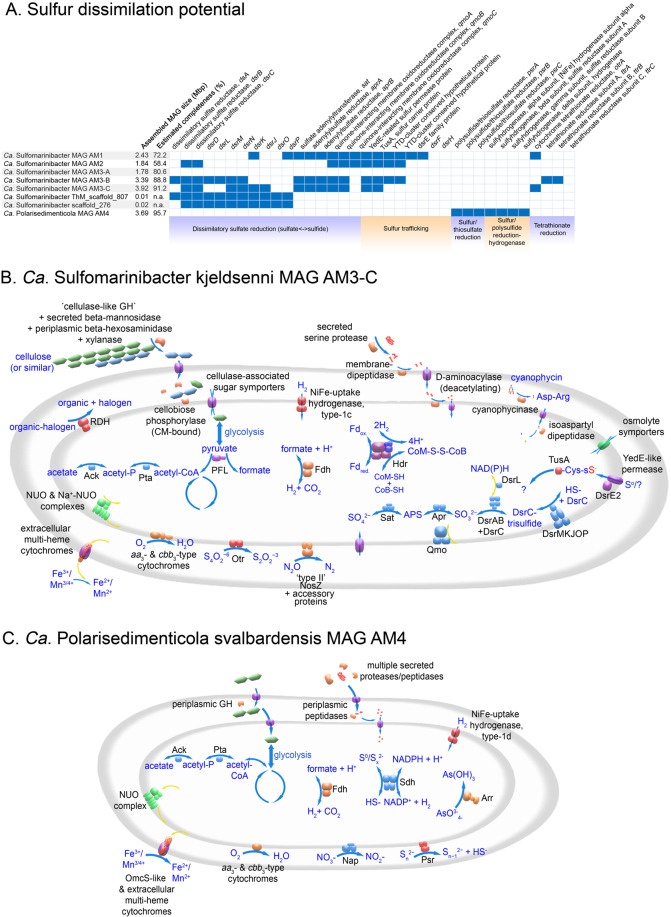
Diverse sulfur-dissimilating potentials predicted among *Ca*. Sulfomarinibacter and *Ca*. Polarisedimenticola MAGs. **A** Presence–absence of genes for sulfur-dissimilating enzymes, and metabolic models of (**B**) *Ca*. Sulfomarinibacter kjeldsenii MAG AM3-C, and (**C**) *Ca*. Polarisedimenticola svalbardensis MAG AM4 suggest different fundamental niches of the two species in marine sediments. GH glycoside hydrolase, RDH reductive dehalogenase homologous enzyme, Ack acetate kinase, Pta phosphotransacetylase, PFL pyrivate-fomate lyase, FDH formate dehydrogenase, Hdr heterodisulfide reductase, NUO NADH dehydrogenase, Otr Tetrathionate reductase, NosZ nitrous oxide reductase, Sat sulfate adenylyltransferase, Apr adenylylsulfate reductase, Qmo quinone-interacting membrane oxidoreductase complex, Dsr dissimilatory sulfate reductase, Nap Periplasmic nitrate reductase, Psr poylsulfide reductase, Sdh Sulfhydrogenase complex, and TusA sulfur-carrier protein.

Phylogenomic analyses showed three MAGs (AM1, AM2, and AM3-A) affiliated with GTDB family “FEB-10” of the class Thermoanaerobaculia (“subdivision 23”) (Fig. 1). We included two additional MAGs in our analyses, i.e., AM3-B and AM3-C, that were highly similar to the AM3-A MAG (>98% ANI), but were classified as redundant during MAG dereplication. We studied them in further detail because: (i) the AM3 MAGs represent the most abundant Thermoanaerobaculia species based on sequence coverage (Supplementary Table 2), (ii) they encoded enzymes of interest not present in MAG AM3-A (Fig. 2A), and (iii) were more complete than MAG AM3-A (Table 1). Comparisons of ANI values suggested the Thermoanaerobaculia MAGs represent three distinct species (<95% ANI) (Supplementary Table 3) [90], all from a novel genus for which we propose the name *Candidatus* Sulfomarinibacter (Supplementary Table 4). The MAG AM3-C represents the type species *Ca*. Sulfomarinibacter kjeldsenii (Supplementary Table 4). MAG AM4 represents the type species of another novel genus affiliated with the GTDB class “Mor1” (“subdivision 22”) (Fig. 1), and for which we propose the name *Ca*. Polarisedimenticola svalbardensis (Table 1 and Supplementary Table 4).

**Table 1:** Summary of Acidobacteriota MAGs retrieved from Svalbard.

MAG	MAG (bp)	Estimated genome size (bp)	GC (%)	Completeness (%)	Contamination (%)	Strain heterogeneity	Number of contigs	GenBank accession
*Ca*. Sulfomarinibacter MAG AM1	2,426,940	3,362,810	63.3	72.2	2.9	40.0	666	JACXVY000000000
*Ca*. Sulfomarinibacter MAG AM2	1,835,749	3,146,099	63.0	58.4	7.7	43.0	828	JACXVZ000000000
*Ca*. Sulfomarinibacter MAG AM3-A	1,779,173	2,206,863	60.9	80.6	1.1	0.0	127	JACXWA000000000
*Ca*. Sulfomarinibacter MAG AM3-B	3,394,205	3,824,456	60.7	88.8	4.3	20.0	533	JACXWB000000000
*Ca*. Sulfomarinibacter kjeldsenii MAG AM3-C	3,921,116	4,316,434	60.9	91.2	3.4	20.0	783	JACXWC000000000
*Ca*. Polarisedimenticola svalbardensis MAG AM4	3,685,148	3,887,504	62.2	95.7	6.0	0.0	180	JACXWD000000000

### Marine Acidobacteriota encode the full dissimilatory sulfate reduction pathway

**Fig. 3:**
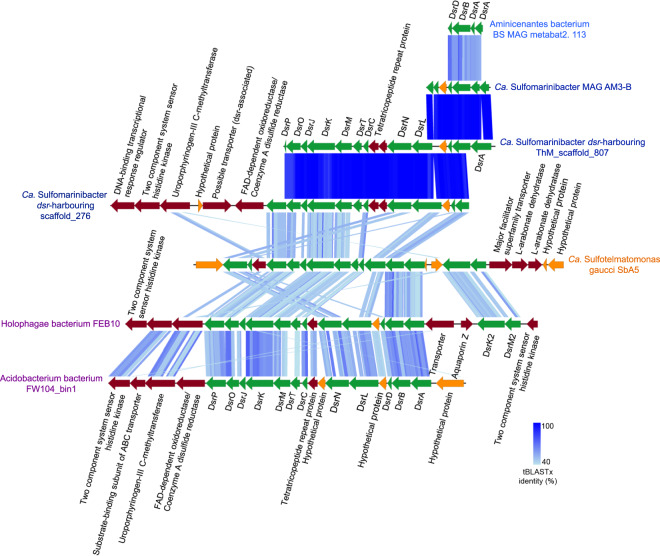
Gene organization of the *dsr* gene cluster in Acidobacteriota. Scaffold names in blue were retrieved from this study. Scaffold names in purple were derived from best BLASTP hits to sequences from this study. *Ca*. Sulfotelmatomonas gaucii SbA5 was retrieved from Hausmann et al. [2]. Green: *dsr*, dark red: other genes, and orange: hypothetical genes. Shaded blue lines indicate degree of sequence similarity as determined by tBLASTx within EasyFig [148], which depicts sequence identity similarities among protein sequences.

Together, the gene content of the *Ca*. Sulfomarinibacter suggests they encode a complete canonical dissimilatory sulfate reduction pathway (Fig. 2A, B and Supplementary Table 5). This includes enzymes required for sulfate activation to APS (Sat) and reduction of APS to sulfite (AprAB, QmoABC), and further reduction of sulfite to sulfide (DsrAB, DsrC, DsrMKJOP, and DsrN) (Fig. 2A, B and Supplementary Table 5). Acidobacteriota *dsr* were also found on scaffolds (up to 20 kb) that were not binned into MAGs, yet had highly similar genes and therefore derive from closely related populations, e.g., >99% *dsrB* nucleotide identity (Fig. 3 and Supplementary Table 5). The unbinned acidobacteriotal contig “ThM_scaffold_807” harbored all *dsr* on one contig (Fig. 3). The predicted DsrC had two conserved cysteine residues critical for respiratory functioning (Supplementary Fig. 2) [91]. Similar to Acidobacteriota MAGs from peatlands and permafrost [2, 12], the marine Acidobacteriota encoded both DsrL and DsrD proteins. DsrL acts as a NAD(P)H:acceptor oxidoreductase for DsrAB [92], while the function of DsrD has not been proven, it is possibly a transcriptional regulator [93]. The DsrL sequences were phylogenetically related to group “DsrL-2” from *Desulfurella amilsiiI* (38% amino acid identity), peatland Acidobacteriota and other subsurface bacteria, and were phylogenetically distinct from group “DsrL-1” of sulfur-oxidizing aerobes (Supplementary Fig. 3A) [94]. The DsrL had conserved YRR-motifs in the NAD(P)H substrate-binding domains that are present in the DsrL-2 group, and absent in DsrL-1 of sulfur-oxidizing aerobes (Supplementary Fig. 3B) [94].

**Fig. 4:**
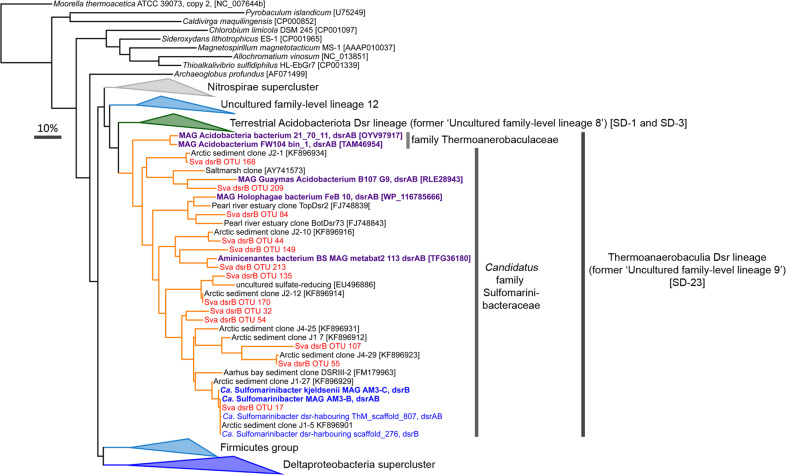
DsrAB uncultured family-level lineage 9 in the DsrAB tree represents members of the Acidobacteriota class Thermoanaerobaculia (subdivision 23). Blue leaves in the DsrAB tree represent MAGs or contigs identified in this study. Red leaves represent the most abundant acidobacteriotal amplicon-derived DsrB sequences identified in this study. Purple leaves represent sequences from MAGs retrieved from public databases. The DsrAB sequences were added to the consensus tree from Müller et al. [36] in ARB. SD, pertaining to “subdivisions” of Acidobacteriota. Numbers in parentheses are Genbank accessions. The scale bar represents 10% sequence divergence.

The DsrAB sequences from the novel Acidobacteriota MAGs and unbinned metagenomic contigs are phylogenetically affiliated with the “Uncultured family-level lineage 9” within the “Environmental supercluster 1”, which is part of the “reductive, bacterial-type DsrAB branch” in the DsrAB tree [36] (Fig. 4). Sequences of “lineage 9” are primarily derived from marine sediments [36]. This lineage is closely related to the “Uncultured family-level lineage 8” that harbors DsrAB sequences from peatland- and permafrost-derived Acidobacteriota of subdivisions 1 and 3 [2, 12]. We also identified several “lineage 9” *dsrA* and/or *dsrB* sequences in Acidobacteriota MAGs from public databases that derived from marine or groundwater environments (Fig. 4). Herein, we refer to this clade as the “Thermoanaerobaculia Dsr lineage”.

### Marine Acidobacteriota use tetrathionate and potentially also other sulfur-cycle intermediates

**Fig. 5:**
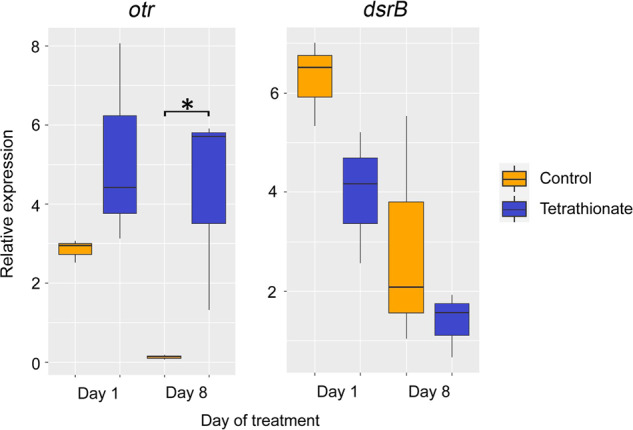
Box plots depicting the expression of *otr* and *dsrB* relative to a housekeeping gene (DNA-directed RNA polymerase, alpha subunit) from *Ca*. Sulfomarinibacter kjeldsenii MAG AM3-C during microcosm experiments with amendments of tetrathionate versus no-amendment controls. Relative expression was determined by rt-qPCR. Expression of *otr* was significantly higher at day 8 (*p* = 0.0488) as determined using a two-tailed *T*-test, and is indicated by an asterisk. Center lines indicate medians; box limits indicate 25th and 75th percentiles as determined by R software; and whiskers extend 1.5 times the interquartile range from the 25th and 75th percentiles.

Several *Ca*. Sulfomarinibacter MAGs encoded *c*-type cytochromes annotated as octaheme tetrathionate reductases (Otr), which was supported by phylogenetic analysis (Supplementary Fig. 4) [95]. The Otr were predicted to be periplasmic and may enable respiration with tetrathionate, a sulfur compound of intermediate oxidation state (“sulfur-cycle intermediate” (SCI)) [31] (Fig. 2B). To provide support that the Otr catalyses tetrathionate reduction and that this occurs within in situ-like conditions, we conducted anoxic microcosm experiments with Svalbard sediments with additions of tetrathionate (500 µM) versus controls without any additions (all contained 28 mM sulfate). We hypothesized that additions of tetrathionate, which has a relatively high redox potential (+198 ± 4 mV versus standard hydrogen electrode (SHE)) [96], would trigger increased expression of *otr* because it is a favorable electron acceptor over sulfate. We thus examined expression of *otr* and *dsrB* of *Ca*. Sulfomarinibacter MAG AM3-C by RT-qPCR analysis of reverse-transcribed mRNA, from multiple times points after tetrathionate additions. Relative expression levels of these transcripts were determined in comparison to the housekeeping gene for DNA-directed RNA polymerase alpha subunit, in order to account for potential changes in cell numbers over time. This showed that transcription of *otr* was upregulated (1.8-fold) in response to tetrathionate at day 1 versus controls (although not significantly), and was significantly upregulated (*p* < 0.0488) 36-fold at day 8. Additionally, the transcription of *dsrB* appeared lower at both days in tetrathionate-amended microcosms versus controls (0.48–0.63-fold), although not significantly (Fig. 5).

“YTD gene clusters” encoding sulfur-trafficking rhodonase-like proteins [97] were identified among *Ca*. Sulfomarinibacter MAGs. Genes for YedE-related permease-like proteins, a DsrE2-like protein, a rhodonase-domain containing sulfur-carrier TusA, and two conserved hypothetical proteins, were present in the YTD gene clusters (Supplementary Table 5). The TusA sulfurtransferase had conserved Cys‐Pro‐X‐Pro sulfane sulfur‐binding domains (Supplementary Fig. 5A). The TusA were phylogenetically most closely related to various TusA from anaerobic Desulfobacterota that are capable of reducing and/or disproportionating inorganic sulfur compounds such as elemental sulfur, sulfite, and/or thiosulfate (Supplementary Fig. 5B). Together, this suggested *Ca*. Sulfomarinibacter are capable of internal trafficking of sulfur, and may use it to reduce and/or disproportionate inorganic sulfur compounds of intermediate redox states.

The marine Acidobacteriota MAGs encoded several Complex-Iron-Sulfur-Molybdoenzyme (CISM) enzymes that may catalyse redox reactions of sulfur compounds. The *Ca*. Sulfomarinibacter MAG AM3-A encoded a putative tetrathionate reductase (TtrA) (Supplementary Fig. 6), and also had an adjacent TtrB (FeS protein) encoded. *A ttrC* encoding a membrane anchor was missing, but the *ttrAB* were situated on the end of the contig and therefore *ttrC* may have been present in DNA that either was not sequenced or on a contig that was not binned. The Ttr complex may provide an additional means to reduce tetrathionate.

*Ca*. P. svalbardensis MAG AM4 had genes for a CISM subunit A enzyme that phylogenetically affiliated with the polysulfide/thiosulfate reductase clade (“Psr”) (Supplementary Fig. 6). Subunits for PsrABC were encoded in a gene cluster, where the terminal reductase PsrA had a TAT-leader peptide for export from the cytoplasm, PsrB had FeS domains for electron transfer between PsrA and PsrC, and the PsrC subunit was predicted to be membrane-bound. This suggested a periplasm location and that the complex may play a role in respiration of sulfur/polysulfide or thiosulfate. Selenite reductases (SrrA) also phylogenetically affiliate with the polysulfide/thiosulfate reductase clade, but conserved rhodonase-like proteins encoded in the gene neighborhood of SrrA are thought to be indicative of selenite-reducing organisms [98], but were absent near *psrABC* in *Ca*. P. svalbardensis MAG AM4.

*Ca*. P. svalbardensis MAG AM4 also harbored a gene cluster encoding four subunits of a sulfhydrogenase complex (Fig. 2A, B and Supplementary Table 5). Similar to the characterized sulfhydrogenase from *Pyrococcus furiosus*, this included two NiFe hydrogenase subunits, as well as two subunits of anaerobic sulfite reductases [99–101]. These complexes can use elemental sulfur or polysulfides as electron sinks when available [101], or act in reverse as hydrogen-evolving hydrogenases during fermentative growth [102].

### Marine Acidobacteriota may respire additional electron acceptors including metals

All MAGs had gene clusters encoding multi-heme *c*-type cytochromes with predicted periplasmic or extracellular locations, as well as associated predicted β-barrel proteins (Fig. 2A, B, and Supplementary Table 5). In known metal-reducing and/or -oxidizing bacteria, extracellular, and periplasmic cytochromes can insert into outer-membrane traversing β-barrel proteins, and transfer electrons through the complexes to/from metals [103, 104]. These gene clusters were syntenous among the MAGs and *Thermoanaerobaculum aquaticum* (Supplementary Fig. 7A), a related hot spring-derived isolate that can anaerobically reduce iron- and manganese-oxides [11]. We therefore propose these cytochromes are likely candidates for facilitating the reduction of metal-oxides by *Thermoanaerobaculum aquaticum*, because no other predicted extracellular cytochromes are encoded by its genome. It is therefore probable that the similar cytochromes encoded by our marine MAGs may also perform this function.

The *Ca*. P. svalbardensis MAG AM4 encoded two additional cytochrome-*c* proteins with similarity to metal-reducing outer-membrane cytochromes (OmcS) from known metal-reducing bacteria, i.e., various Desulfuromonadia (formerly Desulfuromonadales) such as *Geobacter* and *Geopsychrobacter* spp. (Supplementary Table 6) [105, 106]. These cytochromes had six heme-binding sites like characterized OmcS, and were also clustered among genes for predicted periplasmic cytochromes and β-barrel proteins (Supplementary Fig. 7B). They could therefore also potentially exchange electrons with metal-oxides (or other insoluble substrates such as humic-like substances, or other cells) (Fig. 2B, C).

The marine Acidobacteriota MAGs also encoded the potential to reduce various other electron acceptors. Both groups have potential for oxygen reduction through cytochromes (Fig. 2B, C), although we speculate *Ca*. Sulfomarinibacter may reduce oxygen as a defense mechanism, because they otherwise encode various features of strict anaerobes (Supplementary Information). *Ca*. Sulfomarinibacter also encode type-II nitrous oxide reductases (Fig. 2B and Supplementary Fig. 8) and reductive dehalogenases (Fig. 2B and Supplementary Fig. 9), suggesting nitrous oxide and organohalides could be reduced by these enzymes, respectively. *Ca*. P. svalbardensis encodes periplasmic nitrate reductases, although no other genes for enzymes for other steps of denitirification (Fig. 2C and Supplementary Table 5). A putative arsenate reductase is also encoded by *Ca*. P. svalbardensis (Supplementary Fig. 6 and Supplementary Table 4).

### Additional energy conserving mechanisms among marine Acidobacteriota

Electron-bifurcating heterodisulfide reductase complexes were only encoded in *Ca*. Sulfomarinibacter MAGs (Fig. 2B and Supplementary Table 5). These complexes enable flavin-based redox balancing and formation of low-potential electron carriers (i.e., ferredoxin and/or flavodoxin), and are common among strict anaerobes [107, 108]. A high-molecular-weight cytochrome *c*_3_-type protein with a predicted periplasmic location was encoded in *Ca*. Sulfomarinibacter MAG AM1 (Supplementary Table 5). These typically act as periplasmic redox hubs to link electron flows between the periplasm and cytoplasm in SRM [109]. All Acidobacteriota MAGs recovered in this study encoded NADH-ubiquinone oxidoreductase (Nuo) complexes required for energy conservation via respiration (Fig. 2B, C and Supplementary Table 5). Genes for additional sodium-dependent Nuo complexes were also present (Fig. 2B and Supplementary Table 5). Apart from the potential for respiration, some Acidobacteriota MAGs from both *Ca*. Sulfomarinibacter and *Ca*. P. svalbardensis MAG AM4 encoded acetate kinase and phosphate acetyltransferase for fermentation via acetogenesis, or which may act in reverse to facilitate acetate consumption (Fig. 2B, C and Supplementary Table 5).

### Marine Acidobacteriota use diverse nutrient and electron sources

The *Ca*. Sulfomarinibacter AM3 MAGs encoded predicted cellulase A enzymes with signal peptides for export from the cytoplasm (Fig. 2B). They were phylogenetically affiliated with cellulase A from various anaerobic degraders of cellulose and/or plant-derived polysaccharides (Supplementary Fig. 10). A cellobiose phosphorylase was encoded in *Ca*. S. kjeldsenii MAG AM3-C, and had relatively high amino acid identity (63%) to a characterized cellobiose phosphorylase from *Thermotoga neapolitana* [110]. These enzymes catalyse phosphorolysis of cellobiose to ɑ-D-glucose 1-phosphate (G1P) and D-glucose, thereby saving an ATP before entering glycolysis, and are typically used by anaerobic cellulose-degraders [111]. This suggests these organisms have the capacity to anaerobically degrade cellulose, a derivative of cellulose, or a structurally similar compound. Overall, the marine *Ca*. Sulfomarinibacter MAGs encoded few genes for glycoside hydrolases or other carbohydrate active enzymes, i.e., 0.47–0.75% of protein encoding genes encoded glycoside hydrolases (further detailed in Supplementary Information) (Supplementary Table 7). The *Ca*. P. svalbardensis MAG AM4 also encoded few glycoside hydrolases (0.54% of protein encoding genes), with none predicted to be exported to the extracellular environment, and a single endo-1,4-beta-xylanase predicted to be periplasmic (Supplementary Table 5).

Genes for cyanophycinases among *Ca*. Sulfomarinibacter MAGs indicated they may utilize the storage compound cyanophycin as a nutrient (Fig. 2B and Supplementary Table 5). The cyanophycinases had Secretion-signal peptides (Sec-) for export from the cytoplasm, indicating they act on an external substrate and not an internally stored compound. Accordingly, no genes for cyanophycin synthetases were found. An isoaspartyl dipeptidase was encoded in *Ca*. S. kjeldsenii MAG AM3-C, which may enable utilization of the products released by the cyanophycinase, i.e., a dipeptide of aspartate and arginine (Supplementary Table 5). The capacity to catabolically degrade aspartate and arginine was also encoded (Supplementary Table 5).

The *Ca*. Sulfomarinibacter MAG AM3-C may degrade extracellular proteins using two predicted secreted proteases, as well as adjacently encoded peptidases predicted to be membrane-bound (Fig. 2B and Supplementary Table 5). The *Ca*. P. svalbardensis MAG AM4 harbored numerous genes for proteases/peptidases (*n* = 7) that were predicted to be secreted, strongly indicating these bacteria use proteins as nutrients (Fig. 2C and Supplementary Table 5).

Membrane-bound NiFe uptake-hydrogenases were encoded by both *Ca*. Sulfomarinibacter and *Ca*. P. svalbardensis MAGs (Fig. 2B, C and Supplementary Table 5). These may be important for oxidizing environmental hydrogen. The *Ca*. Sulfomarinibacter MAGs encoded “type-1c” NiFe hydrogenases typically found in obligate anaerobes and that are thought to be oxygen sensitive (Supplementary Fig. 11) [112]. The *Ca*. P. svalbardensis MAG AM4 encoded a “type-1d” NiFe hydrogenase, which are typically found in aerobes and facultative anaerobes (Supplementary Fig. 11) [112]. Inspection of best BLASTP hits from the NCBI-nr database to the *Ca*. Sulfomarinibacter NiFe hydrogenase sequences identified various sequences previously shown to be expressed in tidal flat sediments [113]. Formate dehydrogenases encoded among MAGs of both *Ca*. Sulfomarinibacter and *Ca*. P. svalbardensis also suggested formate may be used as an electron donor (Fig. 2B, C and Supplementary Table 5).

### Genome comparisons reveal distinct properties between novel genera and adaptations to marine environments

To compare unique proteins between *Ca*. Sulfomarinibacter and *Ca*. P. svalbardensis, and therefore potential functional differences, we performed reciprocal BLASTP analyses of protein sequence complements from each genus (Supplementary Table 8, and further detailed in the Supplementary Information). In this section, we describe unique proteins encoded in multiple copies (>2 SD above the mean, excluding hypotheticals/unknowns) among each set of unique protein sequences. We hypothesized these might be of special importance for the organisms if they are present in multiple copies (Supplementary Table 8). The whole list of unique proteins are provided in Supplementary Table 8. Among proteins unique to *Ca*. Sulfomarinibacter were: (i) various mobile element proteins (transposases), (ii) rubrerythrins that are known to play roles in detoxification pathways for reactive oxygen species (e.g., H_2_O_2_) in anaerobes [114], (iii) heterodisulfide reductase subunits (described above), (iv) sulfatases that may play a role in removing sulfate groups from organics [115], (v) hydrolases (beta-lactamase super-family) that may play various roles, including hydrolyzing antibiotic compounds. Among those unique to *Ca*. P. svalbardensis were various: (i) peptidases/proteases, (ii) toxin-related proteins, (iii) *c*-type cytochromes, and (iv) tetratricopeptide-repeat-containing proteins that often function in protein–protein interactions [116]. Overall, these comparisons highlight many differences in protein content among the two genera, likely reflecting distinct physiologies and the large phylogenetic distances between the two genera.

Comparisons of encoded protein content of the marine Acidobacteriota MAGs with seven *dsr*-harboring Acidobacteriota MAGs from peatland soil [2] suggested the marine Acidobacteriota encoded unique adaptations to marine settings (Supplementary Information) (Supplementary Fig. 12). These included various predicted transporters/symporters and pumps for ions (e.g., sodium and potassium) and metals/metalloids (e.g., zinc and arsenic) that were unique to the marine MAGs. Genes for a sodium-translocating NADH-quinone oxidoreductase complex, which are used by various marine microorganisms to support respiration and cellular homeostasis [117], were only present in marine MAGs. Symporters for the osmolytes proline, glutamate and glycine, were also only present in marine MAGs.

### Acidobacteriota are prevalent, active, and diverse in marine sediments

**Fig. 6:**
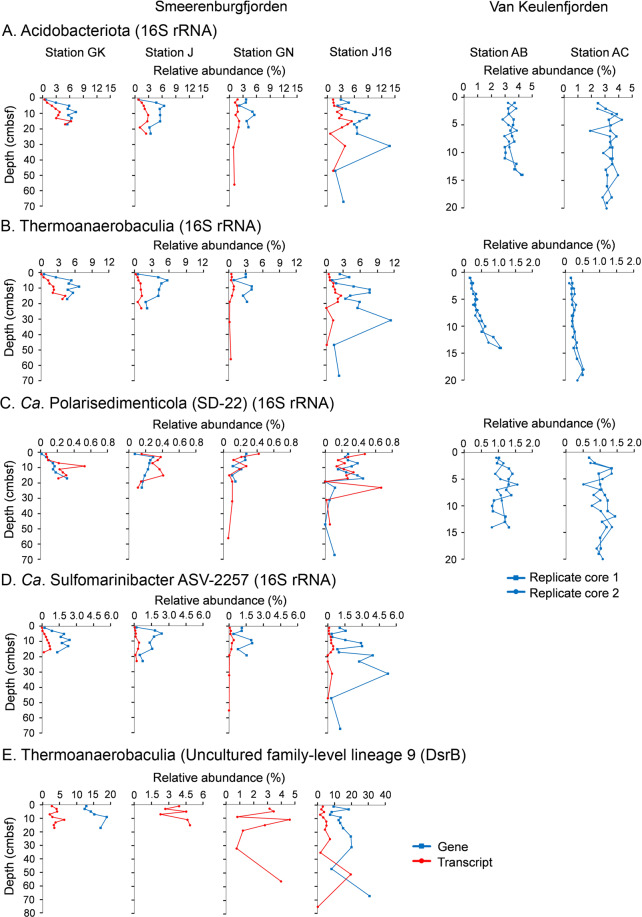
Acidobacteriota are abundant and transcriptionally active in sediments of Svalbard. **A** Relative abundances of phylum Acidobacteriota 16S rRNA genes or transcripts. **B** Relative abundances of class Thermoanaerobaculia 16S rRNA genes or transcripts. **C** Relative abundances of class *Ca*. Polarisedimenticolia 16S rRNA genes or transcripts. **D** Relative abundances of *Ca*. Sulfomarinibacter ASV_2257 16S rRNA genes or transcripts. **E** Relative abundances of Thermoanaerobaculia *dsrB* genes or transcripts. Relative abundances for genes or transcripts are depicted in red or blue, respectively. Smeerenburgfjorden stations GK, J, and GN were sampled in June 2017, and J16 was sampled in July 2016. Replicate cores from Van Keulenfjorden stations AB and AC are derived from Buongiorno et al. [40].

Amplicon sequencing of 16S rRNA genes and transcripts was performed from cores of Smeerenburgfjorden sediments (Fig. 6A–D and Supplementary Fig. 13). General features of these sediments include: (i) high sulfate reduction rates that peak near 100 nmol SO_4_^–2^ cm^−3^ d^−1^ around 5–6 cmbsf and slowly decline with depth, (ii) iron reduction in the top 5 cm of sediments of station J and GN, (iii) higher amounts and deeper accumulation of dissolved iron (>250 µM) from dissimilatory iron reduction in sediments of station GK, and (iv) free sulfide accumulates below 10 cm in station J and GN sediments [38, 41, 113]. Overall, the sequencing results revealed Acidobacteriota had an average relative abundance of 4.5 ± 2.2% (Fig. 6A and Supplementary Fig. 13). Thermoanaerobaculia-affiliated sequences were the most dominant of any Acidobacteriota (Fig. 6B), and reached the most abundant (11%) genus-level clade of Bacteria at 31 cmbsf in Station J (sampled in 2016). The same clade was on average the fourth most abundant genus-level clade in the same core (averaged 4.5 ± 2.8%). 16S rRNA transcripts of Thermoanaerobaculia-affiliated sequences were below 0.5% relative abundances in the surface sediments (0–1 cmbsf) of Smeerenburgfjorden cores (Fig. 6B). At station GK, Acidobacteriota 16S rRNA transcripts reached 6% relative abundance at 15 cmbsf (Fig. 6B).

We also examined Acidobacteriota 16S rRNA genes from metal-rich Van Keulenfjorden sediment cores (Fig. 6A–D), from a previously published study [40]. Sediments from these cores are characterized by very high concentrations of dissolved iron (up to >500 µM) and manganese (>100 µM) that persist over tens of centimeters of depth, while exhibiting low sulfate reduction rates (<12 nmol SO_4_^–2^ cm^−3^ d^−1^) [38, 41]. The sequence analyses showed *Ca*. Polarisedimenticolia related sequences were the most prominent Acidobacteriota, reaching 1.5%, and averaging 1.1 ± 0.21% of communities in four cores (Fig. 6C). Members of the Thermoanaerobaculia were in much lower abundances (0.3 ± 0.2% average overall), although they reached 1.1% in deeper sections of core AB (Fig. 6B).

Mapping of metagenomic reads to the Acidobacteriota MAGs supported the general distribution trends from 16S rRNA amplicon analyses, i.e., that Thermoanaerobaculia were abundant in Smeerenburgfjorden sediments and *Ca*. Polarisedimenticolia were more abundant in Van Keulenfjorden sediments (Supplementary Table 2, and further detailed in Supplementary Information).

Phylogenetic analysis of 16S rRNA genes from Smeerenburgfjorden sediments revealed diverse Acidobacteriota sequences (Supplementary Fig. 14). Examination of the taxonomy of Acidobacteriota 16S rRNA sequences derived from marine sediments in the SILVA database also revealed that Thermoanaerobaculia (subdivision 23) and *Ca*. Polarisedimenticolia (subdivision 22) are among the most prominent Acidobacteriota lineages in marine sediments (Supplementary Fig. 15, and further detailed in Supplementary Information).

**Fig. 7:**
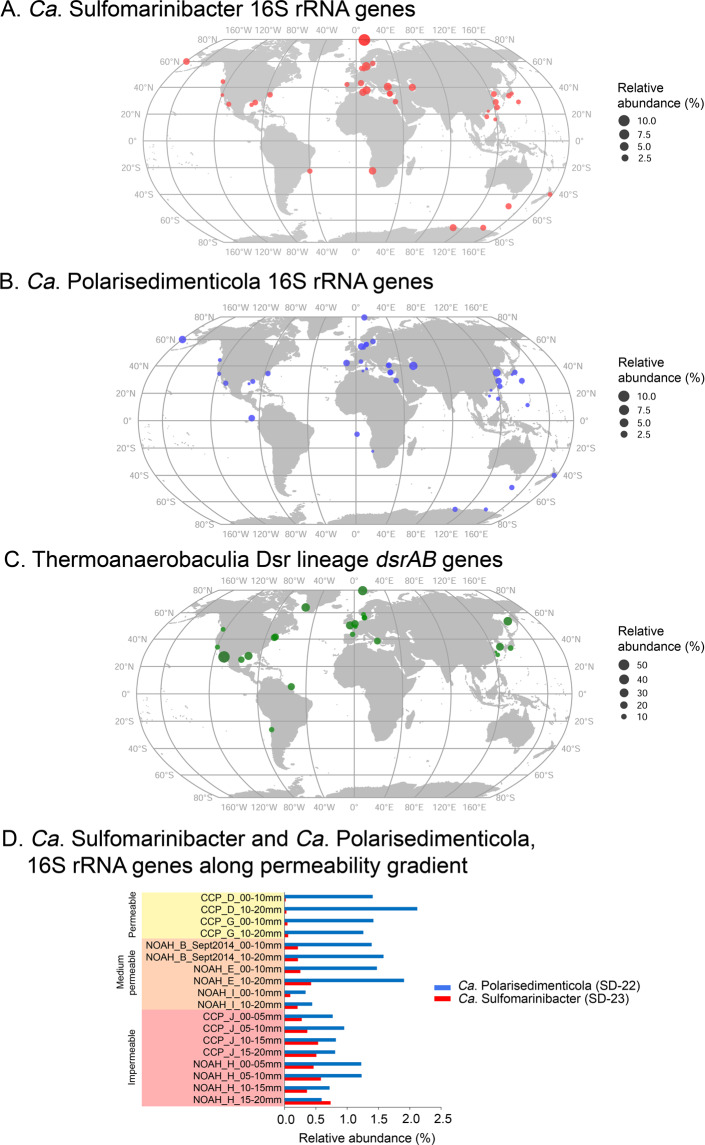
Acidobacteriota 16S rRNA and *dsrAB* genes are prevalent in marine sediments worldwide. Global maps depicting presence and relative abundances of: **A** *Ca*. Sulfomarinibacter 16S rRNA genes; **B** *Ca*. Polarisedimenticola 16S rRNA genes; **C** Thermoanaerobaculia *dsrA*/*B* genes. **D** Relative abundances of *Ca*. Sulfomarinibacter and *Ca*. Polarisedimenticola 16S rRNA genes in sediments from the North Sea that exhibit a permeability gradient (Probandt et al. [136]), and that were reanalysed in this study. Relative abundances presented from clone library-based studies were calculated from all sequences belonging to each study. Relative abundances presented from amplicon sequencing-based studies (Short Read Archives) were derived from the highest relative abundance found in any individual sample per study. As exception was that an average relative abundance of Thermoanaerobaculia *dsrB* was calculated from all *dsrB* sequences from the Northwestern Gulf of Mexico study (23 surface sediment samples) [147]. List of studies/datasets used are listed 10.6084/m9.figshare.14369657.v1.

Analyses of 16S rRNA gene sequences among publically available datasets derived from diverse locations further showed members of the *Ca*. Sulfomarinibacter and *Ca*. Polarisedimenticolia are prevalent in marine sediments worldwide (Fig. 7A, B). Among the 40 16S rRNA gene study datasets where either *Ca*. Sulfomarinibacter and/or *Ca*. Polarisedimenticolia were identified, they averaged 1.6% and 1.2% of sequences, respectively. Notably, *Ca*. Sulfomarinibacter sequences were absent from sediments of abyssal and hadal trenches [114–116], and from sediments underlying mid-oceanic sites/oceanic gyres [117, 118], i.e., sediments with low organic inputs and where oxygen typically penetrates centimeters to tens-of-centimeters into sediments [119, 120]. Only *Ca*. Polarisedimenticolia sequences were identified at several of these deep water sites (Fig. 7B). Analyses of sequence data from surface sediments (0–2 cmbsf) of the North Sea that exhibit different permeability properties [121], revealed *Ca*. Sulfomarinibacter sequences were in very low relative abundances in the most permeable sites (<0.06%), yet reached a maximum of 0.7% in the impermeable sediments (Fig. 7D). In contrast, *Ca*. Polarisedimenticolia reached up to 2% relative abundances in the permeable sediments (Fig. 7D). These two groups therefore appear to have contrasting tolerances for oxygen.

### Acidobacteriota actively express dsrB and are prominent dsrAB-harboring bacteria in marine sediments

Sequencing of *dsrB* genes and transcripts from Smeerenburgfjorden sediments revealed that Acidobacteriota *dsrB* averaged 13 ± 6.6% of all *dsrB* (DNA-derived) sequences, and 4 ± 2% of *dsrB-*transcripts (cDNA-derived) (Fig. 6E and Supplementary Fig. 16). Acidobacteriota *dsrB* sequences were the second most abundant group after Desulfobacterota *dsrB*, which dominated the sediments and averaged 75 ± 6% in relative abundance (Supplementary Fig. 16). Acidobacteriota *dsrB* reached a maximum of 19% at station GK and 31% at station J. The most abundant Acidobacteriota *dsrB-*OTU-17 was 100% identical (over 321 nucleotides) to *dsrB* from *Ca*. Sulfomarinibacter AM3-B MAG (Fig. 4). Amplicon-derived DsrB sequences that affiliated with the DsrB from marine Acidobacteriota MAGs were phylogenetically diverse and spread through-out the “Thermoanaerobaculia Dsr clade” (Fig. 4).

Analyses of publically available *dsrAB* sequence datasets (*n* = 24) revealed sequences of the Thermoanaerobaculia *dsr-*lineage were widespread (Fig. 7C) and averaged 15% of all *dsrAB* sequences analysed (*n* = 14,077 classified sequences). They reached a maximum of 54.3% in hydrothermally influenced sediments of the Guaymas Basin [118]. From all datasets analysed, sequences from the Thermoanaerobaculia *dsr-*lineage were the second most abundant *dsrAB* amplified from marine sediments after sequences of the Desulfobacterota.

### Description of novel Acidobacteriota *Candidatus* taxa

Based on their unique phylogeny, predicted metabolic properties, CARD-FISH visualized cells of Thermoanaerobaculia (thin rods present in three different sites, see Supplementary Fig. 17) and relatively complete MAGs, we propose the following new *Candidatus* taxa of Acidobacteriota (Supplementary Table 4):

class Thermoanaerobaculia (subdivision 23)

 order Thermoanaerobaculales

  fam. nov. *Ca* Sulfomarinibacteraceae (GTDB family FEB-10)

   gen. nov. *Ca*. Sulfomarinibacter

    sp. nov. *Ca*. Sulfomarinibacter kjeldsenii sp. nov. MAG AM3-C

       *Ca*. Sulfomarinibacter sp. MAG AM1

       *Ca*. Sulfomarinibacter sp. MAG AM2

class nov. *Ca*. Polarisedimenticolia (GTDB class Mor1, subdivision 22)

 ord nov. *Ca*. Polarisedimenticolales (GTDB order Mor1)

  fam. nov. *Ca*. Polarisedimenticolaceae (GTDB family Mor1)

   gen. nov. *Ca*. Polarisedimenticola

    sp. nov. *Ca*. Polarisedimenticola svalbardensis MAG AM4.

## Discussion

This study provides the first insights into the genomes and metabolic potential of abundant Thermoanaerobaculia from marine sediments, and new insights into the metabolisms of *Ca*. Polarisedimenticolia (Acidobacteriota subdivision 22 or GTDB class Mor1). Most notably, we revealed that MAGs from both of the major lineages of Acidobacteriota from marine sediments have capabilities to dissimilate various inorganic sulfur compounds.

Genes for the full dissimilatory sulfate reduction pathway provided the first direct link between genomes of marine sediment Acidobacteriota and DsrAB sequences of the previously undescribed “Uncultured family-level lineage 9” clade (here named “Thermoanaerobaculia Dsr lineage”). In addition to being abundant and actively transcribed in Svalbard sediments as shown here, our analysis of *dsrAB* sequences from various sediment sites around the world further revealed Acidobacteriota are the next most abundant *dsr*-harboring lineage outside of Desulfobacterota in marine sediments in general. Together, this indicates Acidobacteriota are a widespread and prominent group of inorganic sulfur-dissimilating microorganisms in marine sediments, and therefore likely make significant contributions to the sulfur-cycle in global marine sediments.

While enzymes of the dissimilatory sulfate reduction pathway are widely used for anaerobic reduction of sulfite/sulfate [35], some organisms can use them in reverse for the oxidation of reduced sulfur compounds [122], or for disproportionation of sulfur compounds [123, 124]. Because no enzymes are currently known that distinguish these different metabolisms, discerning sulfur metabolisms based on genomic data requires careful interpretation [123, 124]. For instance, the *Ca*. Sulfomarinibacter MAGs encode DsrL, which was previously thought to be exclusively found in sulfur-oxidizing bacteria [92]. However, recent work showed DsrL can function in a reductive manner in biochemical assays [92, 94], and was highly expressed during reductive sulfur- and thiosulfate-respiration by *Desulfurella amilsii* [92, 125]. The DsrL of *Ca*. Sulfomarinibacter contained putative NADP(H)-binding domain structures that may enable coupling of NADPH as electron donor to sulfite reduction [94], as well as phylogenetic relatedness with DsrL of *Desulfurella amilsii*. Together, this indicates the DsrL of *Ca*. Sulfomarinibacter has potential to facilitate a reductive pathway.

The *Ca*. Sulfomarinibacter MAGs encoded rhodonase-like TusA and DsrE2, which act as sulfur-trafficking proteins in reverse-Dsr harboring sulfur-oxidizing bacteria, i.e., they help deliver sulfur to DsrABC for oxidation [126]. Interestingly, the “YTD gene clusters” that encode these enzymes are also common in genomes of anaerobic elemental sulfur-reducing and/or -disproportionating bacteria that have Dsr, and are suggested to be genetic indicators for disproportionation potential among these anaerobes [97]. The TusA proteins from *Ca*. Sulfomarinibacter were most closely related to TusA from various anaerobic sulfur-reducing and -disproportionating Desulfobacterota (Supplementary Fig. 5A). This suggested *Ca*. Sulfomarinibacter could reduce and/or disproportionate elemental sulfur, or possibly other sulfur compounds that can be trafficked by TusA, like thiosulfate [127]. Indeed, the ability to disproportionate sulfur compounds is common among sulfate-reducing Desulfobacterota [128]. Elemental sulfur is often the most abundant sulfur-cycle intermediate (SCI) in marine sediments [129], and was measured in sediments from Smeerenburgfjorden up to 0.15 wt% of total sulfur [39]. Overall, the gene content of *Ca*. Sulfomarinibacter MAGs indicated flexible dissimilatory sulfur metabolisms that may be dictated by and/or switch under different biogeochemical and redox conditions.

Results indicated *Ca*. Sulfomarinibacter likely use the dissimilatory sulfate reduction pathway in a reductive direction in most depths of the sediments studied. Firstly, Acidobacteriota were relatively abundant and expressed *dsrB* in deeper (>15–75 cmbsf), strictly anoxic sediment layers of Smeerenburgfjorden. These sediments depths lack electron acceptors that could sustain these abundant populations growing via biological oxidation of sulfides, i.e., oxygen, nitrate or oxidized metals [31, 130]. In Station J sediments, oxygen and nitrate are depleted within millimeters-to-centimetres of the surface [131, 132], and sulfide oxidation facilitated by Fe(III) is negligible [41]. An alternative possibility is that cryptic biogeochemical cycling could sustain sulfide oxidation, i.e., fast consumption and production of low concentrations of sulfides and oxidants [133]. Nevertheless, it remains unproven whether biological sulfide oxidation occurs in deep sediments that lack measurable concentrations of required oxidants [28]. On the other hand, the relative abundances of Acidobacteriota peaked in subsurface zones around 5 cmbsf in Station J sediments, where sulfate reduction rates also peak [41, 134]. In another study, Acidobacteriota 16S rRNA gene relative abundances were also highly correlated with sulfate reduction rates in sediments from Greenland [37]. These associations therefore point toward an active role in the reduction and/or disproportionation of sulfur compounds of various oxidation states by *Ca*. Sulfomarinibacter in marine sediments.

Our results also suggested marine Acidobacteriota have potential to reduce various inorganic sulfur compounds independent of the Dsr pathway. Our tetrathionate-amended microcosm experiment suggested *Ca*. Sulfomarinibacter use tetrathionate as an electron acceptor via cytochromes because genes for *otr* increased expression after tetrathionate was added to microcosms (Fig. 5). Although some expression of *otr* was determined in the control at day 1, possibly as a result of expression in the starting sediments, expression of *otr* was nearly undetectable in controls at day 8, but remained high in tetrathionate-amended microcosms. Overall, these results support the roles of these enzymes in tetrathionate reduction under in situ-like conditions. This is noteworthy because these enzymes were only previously shown to perform this function during biochemical assays [135], i.e., their utilization under in situ-like conditions was unknown. The ability to utilize SCI, e.g., tetrathionate or elemental sulfur/polysulfides/thiosulfate, could be important in sediment zones where SCI might be generated from sulfides reacting with available oxidants [41].

The *Ca*. Sulfomarinibacter MAGs indicated they could respire oxygen using terminal *cbb*_3_- or *aa*_3_-type cytochromes, although we speculate these may instead be used for defense against oxygen. This is because they encoded many characteristics of obligate anaerobes, and they appear to prefer subsurface and impermeable sediments where oxygen is absent or scarce (Supplementary Discussion). We also hypothesize that the different redox metabolisms of *Ca*. Sulfomarinibacter and *Ca*. Polarisedimenticola may explain their different abundances among Svalbard fjord sediments with different biogeochemical properties (Supplementary Discussion). That is, the *Ca*. Sulfomarinibacter may be adapted to low redox environments, and are thus more abundant in the reduced (visibly black), sulfidic subsurface sediments of Smeerenburgfjorden. In comparison, the *Ca*. P. svalbardensis MAG had additional genes to utilize high-potential electron acceptors such as oxygen, nitrate and oxidized metals, and these organisms may thus be better adapted to the more high redox, metal-rich sediments of Van Keulenfjorden (visibly reddish-orange). This was also supported by our reanalyses of 16S rRNA genes from a sediment permeability gradient in the North Sea [136], which showed *Ca*. Sulfomarinibacter prefer impermeable sediments where oxygen penetration is limited, while the *Ca*. Polarisedimenticola thrive in permeable sediments where oxygen is available.

If members of the *Ca*. Sulfomarinibacter are indeed SRM, a question arises regarding how they co-exist with dominant sulfate-reducing Desulfobacterota populations, as both can apparently use hydrogen, acetate or formate as substrates. However, we identified genes for use of several organic substrates that may enable *Ca*. Sulfomarinibacter to occupy a distinct nutrient niche. Complex carbohydrates such as cellulose (or structurally similar compounds) could be used. Carbohydrates are not used by most known isolated Desulfobacterota SRM [35]. Plant-derived molecules could stem from terrestrial run-off, which is a major source of organic carbon to arctic sediments [137, 138] and to coastal marine systems in general [139]. Additionally, various marine algae are known to produce cellulose [140]. The predicted ability to utilize cyanophycin could also facilitate a unique nutrient niche. Cyanophycin is a multi-L-arginyl-poly-L-aspartic acid, commonly produced by cyanobacteria as a storage compound [141, 142]. Indeed, few organisms are known to use cyanophycin anaerobically [143], and no anaerobes are known from marine sediments.

*Ca*. Polarisedimenticola svalbardensis appeared to have a high propensity for the degradation of proteins, which was indicated by a suite of predicted secreted peptidases. A related Mor1 Acidobacteriota genome (GCA_001664505.1) (Fig. 1) was recovered as a bacterial co-inhabitant of a cyanobacterial enrichment culture from seawater, suggesting it used organic material/necromass from the primary-producing cyanobacterium [144]. *Ca*. Polarisedimenticola may therefore contribute to protein degradation in marine sediments, where proteinaceous organics comprise a large proportion (~10%) of available organic matter [145].

In summary, the genome-encoded dissimilatory sulfur metabolisms, the high abundances and activity of *Ca*. Sulfomarinibacter in the sulfidic zones of Svalbard sediments, as well as prominence in global marine sediments, suggests these novel Acidobacteriota of the class Thermoanaerobaculia (subdivision 23) are important players in the biogeochemical sulfur cycles of marine sediments. Our data also indicated that *Ca*. Sulfomarinibacter thrive largely via anaerobic metabolisms with the capability to use various other electron acceptors with different redox potentials, including biogeochemically relevant metal-oxides. Additionally, we show that *Ca*. Polarisedimenticola svalbardensis, a member of a different class of Acidobacteriota (subdivision 22), has the genetic potential for protein degradation and for metabolisms driven by high redox potential electron acceptors such as oxygen, nitrate and metal-oxides.

## Supplementary information

Supplementary information - Methods, Results and Discussion

Supplementary Tables 1–8

Supplementary Figures 1–17
