# Supplementary material for: Novel taxa of Acidobacteriota implicated in seafloor sulfur cycling
Source: ISME J. 2021 May 12;15(11):3159–80. doi: 10.1038/s41396-021-00992-0 (PMC8528874; doi:10.1038/s41396-021-00992-0)
Supplement: Supplementary file 1 — Supplementary information - Methods, Results and Discussion [file 41396_2021_992_MOESM1_ESM.docx]

**Supplementary information:**

**Novel taxa of Acidobacteriota implicated in seafloor sulfur cycling**

Mathias Flieder, Joy Buongiorno, Craig W. Herbold, Bela Hausmann, Thomas Rattei, Karen G. Lloyd, Alexander Loy, Kenneth Wasmund.

# Supplementary Materials and Methods

### Sample collection

For sampling sediments from Svalbard, Norway, the following was performed. HAPS core samples were subsampled onboard using a plastic subcorer with 5 cm width and pre-drilled ports every 1 cm. The subsamples were taken from the ports with 3 ml syringes (c. 1 ml sediment), added to 2 ml tubes and flash frozen in a dry-shipper pre-cooled with liquid nitrogen. Rumohr cores were subsampled on land within 48 hrs with 3 ml syringes (*c*. 1 ml sediment) from pre-drilled ports and flash frozen in a pre-cooled dry-shipper. All samples were transported in the cooled dry-shipper and stored at -80°C in the laboratory until nucleic acid extractions. Methods for sediment samples collected previously for 16S rRNA amplicon sequencing from Van Keulenfojorden, were described previously by Buongiorno *et al*. [[1]](https://paperpile.com/c/F1FXsp/QNdua). For metagenomic DNA sequencing, additional samples of sediments (5 ml) were added to 15 ml tubes using sterile spatula and immediately snap-frozen as above. Sediments for incubation experiments were taken from HAPS cores by carefully removing the top 5 cm of sediment with clean spatulas, then collecting 5-10 cmbsf of sediment directly to autoclaved glass jars (filled completely with sediment to exclude as much oxygen as possible), capped and sealed tightly with tap. These samples were stored ship-board (temperatures never exceeded 7°C in the field), wrapped with ice-packs during transport (<48 hrs), or at 4°C when in the laboratory.

Additional samples for non-quantitative microscopy were taken from tidal flat sediments of Aveiro Lagoon, Portugal (40°34.14N 8°45.10W) in July 2019, and from Kristineberg station near Fiskebäckskil, Sweden (58°24.95N, 11°44.50E) in October 2019. For sampling sediments from Aveiro Lagoon, Portugal, the following was performed. Samples (dark-black sandy-mud) were taken from the exposed tidal flat during low tide, approximately 5 m onto the tidal flat from the high tide mark. Visibly oxidized surface sediments (*c*. 5 cm) were scrapped-off with a clean spatula, then 5-10 cmbsf of black sediment was filled into clean glass jars, capped and sealed tightly with tape. The samples were wrapped with ice-packs during transport (<72 hrs), and stored at 14°C back in the laboratory. Sediments from near Kristineberg station of Gullmarsfjorden, Sweden, were collected by a trawling boat. Roughly 30 cm of the top sediment was collected by a grab core. The samples were stored at 4°C in 50 ml falcon tubes until transport to Vienna. Falcon tubes were transported to Vienna on ice packs and stored at 4°C in the laboratory.

### Microcosm incubations used for metagenomic sequencing

Microcosms from which some of the metagenomic samples were derived (Supp. Table 1), were prepared as above, except samples collected from 2016 were used. These were also from 5-10 cmbsf of Station J, Smeerenbergfjorden. Incubations were set-up as described in the section of the main text ‘*Microcosm incubations with tetrathionate additions’*, except treatments included only additional sulfur compounds (i.e., no yeast extract): i) tetrathionate (500 µM); ii) tetrathionate (500 µM) + molybdate (28 mM); iii) thiosulfate (500 µM) + molybdate (28 mM). Samples (500 µl) were collected after 9 days of incubation at 4°C.

### Nucleic acid extraction using phenol/chloroform extraction method

Extractions of total nucleic acids using the phenol/chloroform extraction method were performed as previously described [[2]](https://paperpile.com/c/F1FXsp/n37i2). All centrifugation steps were conducted at 20000 g and 4°C, and tubes were kept on ice between steps. Briefly, sediment samples (500 µl) were placed in 2 ml tubes, and 375 µl buffer PB (10 mM sodium phosphate buffer, pH 8.0), 125 µl buffer TNC (500 mM Tris-HCl, 100 mM NaCl, 10% w/v cetrimonium bromide [CTAB]), and 500 µl Roti-Phenol (Carl Roth GmBH) were added, mixed, and transferred to ‘Lysis tube E’ bead beating tubes (MP Biomedicals). Tubes were bead beaten with a FastPrep®-24 bead beater (MP Biomedicals) for 30 sec on speed 6, centrifuged for 3 min, and the supernatant (*c*. 700 µl) was transferred to new 2 ml tubes. Next 700 µl of phenol/chloroform/isoamyl-alcohol (25:24:1; Carl Roth GmBH) was added, mixed by inverting several times and centrifuged for 3 min. The supernatant (*c*. 600 µl) was transferred to a new 2 ml tube, then 600 µl of chloroform/isoamyl-alcohol (24:1; Carl Roth GmBH) was added, mixed and centrifuged for 3 min. The supernatant (*c*. 500 µl) was transferred to a 1.5 ml non-stick tube (Eppendorf), 1 ml cold polyethylenglycol (PEG) solution and 2 µl glycogen (20 mg ml^-1^, RNA grade, Thermo Fisher) were added, mixed carefully and centrifuged for 30 min. The supernatant was discarded and the pellet washed with 175 µl cold 80% molecular grade ethanol and centrifuged for 5 min. The supernatant was discarded, the pellet air-dried for 5 min, resuspended in 100 µl of low TE buffer (1 mM Tris-HCl, 0.01 mM EDTA, pH 8) and stored at -80°C.

### Metagenome sequencing and genome binning

DNA extracted by the Vienna group was sheared using the Covaris microTUBE to 300 bp average size, and barcoded using the NEBNext® Ultra™-II DNA Library Prep Kit for Illumina (New England Biolabs), following the manufacturer’s guidelines.

Sequence reads from samples processed by the Vienna group were trimmed to remove low quality bases (phred cut-off 15, minimum length 50 bp) using a python script, and Illumina adapters were removed using AdapterRemoval using default settings [[3]](https://paperpile.com/c/F1FXsp/Itilp). Sequencing reads processed by the Knoxville group were trimmed using Trimmomatic [[4]](https://paperpile.com/c/F1FXsp/oYwJ0), with a sliding window of 10 and a phred cut-off score of 28 for all reads >90 bp. Reads from samples with especially high read counts (Supp. Table 1) were normalised to a maximum coverage of 100X using BBNorm (target=100 min=5) (version 37.61 of BBMap) [[5]](https://paperpile.com/c/F1FXsp/YBqht).

### Extended annotations and in silico analyses of inferred proteins

Subcellular locations of protein sequences were predicted with PSORTb with the ‘Gram-negative’ option (version 3.0) [[6]](https://paperpile.com/c/F1FXsp/5gFc7). Signal peptides were detected with the PRED-TAT server using the ‘New model (using dipeptides)’ option [[7]](https://paperpile.com/c/F1FXsp/LFrUH), and/or the DeepSig web server [[8]](https://paperpile.com/c/F1FXsp/Jg0zX). Transmembrane helixes were predicted with the TMHMM web server [[9]](https://paperpile.com/c/F1FXsp/MhfPX). Synteny plots of gene organisations were produced using EasyFig [[10]](https://paperpile.com/c/F1FXsp/QZfqg). FeGenie (v20191003) [[11]](https://paperpile.com/c/F1FXsp/GQtF5) analysis was used with default parameters and the ‘-hmm_lib’ option, to identify proteins potentially involved in redox reactions with metal oxides. Potential β-barrel proteins were checked using PRED-TMBB2 server using default settings, i.e., ‘MSA-version (1 sequence)’ for mode, and ‘HMM (original version)’ for algorithm [[12]](https://paperpile.com/c/F1FXsp/3yy4X). Proteins with similarity to carbohydrate-active enzymes were detected using the dbCAN2 server, using the ‘HMMER’ search option [[13]](https://paperpile.com/c/F1FXsp/RrVaH).

***Comparisons of gene content among MAGs***

Comparisons of protein sequence content among the Acidobacteriota MAGs recovered in this study was performed using BLASTP with an e-value of 10^-10^, whereby all protein sequences from either genus were compared against all proteins from the other genus, in a reciprocal manner. Prior to BLASTP, protein sequences from the five *Ca.* Sulfomarinibacter were dereplicated using CD-HIT web server [[14]](https://paperpile.com/c/F1FXsp/v4kYI) using a 90% sequence identity cut-off and default parameters.

Comparisons of protein sequence contents among the various marine and peatland-derived MAGs were performed using OrthoFinder (v2.4.1) to find ortholog groups (OG), using default settings and the ‘diamond’ search option [[15]](https://paperpile.com/c/F1FXsp/03IV3). OGs unique to either of the groups of MAGs being compared were identified by manually sorting present or absent OGs from the ‘Orthogroups’ output table in Excel (Microsoft). Proteins from OGs present in one group of MAGs and absent in the other group of MAGs, were classified as unique. Protein sequences were then extracted for each unique OG, i.e., the first protein sequence listed in the OG was used as a representative for further analysis steps. Functional categories of proteins unique to groups of MAGs then compared by the eggNOG-mapper server by mapping to Clusters of Ortholog Groups database using default settings [[16]](https://paperpile.com/c/F1FXsp/hN0qe).

### Amplicon sequencing and analyses

PCR primers 515F and 806R were modified with a linker sequence [[17]](https://paperpile.com/c/F1FXsp/JMNIC) and barcoded (8 cycles) with a unique dual (UD) setup. First-step PCRs for 16S rRNA genes were performed in triplicate (12.5 μl vol per reaction) with the following conditions: 1X DreamTaq Buffer (Thermo Fisher), 2 mM MgCl_2_ (Thermo Fisher), 0.2 mM dNTP mix (Thermo Fisher), 0.2 µM of forward and reverse primer each, 0.08 mg ml^-1^ Bovine Serum Albumin (Thermo Fisher), 0.02 U Dream Taq Polymerase (Thermo Fisher), and 0.5 µl of DNA template. Conditions for thermal cycling were: 95°C for 3 min, followed by 30 cycles of 30 sec at 95°C, 30 sec at 52°C and 50 sec at 72°C, and finally 10 min at 72°C. Triplicates were combined for barcoding. Barcoding, library preparation and Illumina MiSeq sequencing (using MiSeq Reagent Kit v3 chemistry with 300 bp paired-end mode) was performed by the Joint Microbiome Facility (Vienna, Austria) under project ID JMF-1908-1). Barcoded samples were purified and normalized over a SequalPrep™ Normalization Plate Kit (Invitrogen) using a Biomek® NXP Span-8 pipetting robot (Beckman Coulter), and pooled and concentrated on columns (Anlaytik Jena). Indexed sequencing libraries were prepared with the Illumina TruSeq Nano Kit as described previously [[17]](https://paperpile.com/c/F1FXsp/JMNIC), and sequenced in paired-end mode (2× 300 bp; v3 chemistry) on an Illumina MiSeq following the manufacturer’s instructions. The workflow systematically included four negative controls (PCR blanks, i.e., PCR-grade water as template) for each 90 samples sequenced.

Amplification of *dsrB*-genes or transcripts (cDNA) was done using the primers DSR-1762Fmix and DSR-2107Rmix [[18]](https://paperpile.com/c/F1FXsp/WRvtI) including a head sequence for barcoding (as per barcoding of 16S rRNA amplicons). PCR was performed under the same conditions as described above with thermal cycling conditions: 95°C for 3 min, followed by 40 cycles of 30 sec at 95°C, 30 sec at 60-50°C (touch-down), 60 sec at 72°C, and finally 10 min at 72°C. The touch-down approach was used during the first 10 cycles starting at 60°C and decreasing 1°C each cycle. Barcoding PCR (25 µl vol per reaction) of the *dsrB*-amplicons was performed with individual barcodes of 0.4 µM concentration with the following thermal cycling conditions: 95°C for 3 min, followed by 10 cycles of 30 sec at 95°C, 30 sec at 52°C, 50 sec at 72°C, and finally 72°C for 2 min. DNA was purified using the Zymo ZR-96 DNA Clean-Up Kit (Zymo Research) and quantified using the Quant-iT^TM^ PicoGreen® kit (Thermo Fisher). Pooled samples were sent to Microsynth AG for MiSeq sequencing with 2× 300 bp paired-end mode.

### Primer design and primer specificity checks for RT-qPCR

Novel primers were designed for genes of MAG AM3-C, i.e., for the octaheme tetrathionate reductase gene, the *dsrB*-gene, and the DNA-directed RNA polymerase alpha subunit as a housekeeping gene. The online tool Primer 3 [[19]](https://paperpile.com/c/F1FXsp/GssgT) was used as default except for primer length which was set for 20 +/-1, primer T_m_ was set at 60°C +/-1, GC content was set to 50 +/-5, Max Poly X was set to 3 and the product size range was set to 100-200 bp. Also a manual inspection was done using MEGA 7 [[20]](https://paperpile.com/c/F1FXsp/bw2bI) to check if the designed primers discriminate against closely related outgroups.

Initial PCR were performed on appropriate DNA samples with newly designed primers in triplicates and checked on a 0.8% agarose gel for any visible primer dimer or double bands. Triplicates were combined and purified with the QIAquick PCR purification kit (QIAGEN). The PCR products were transformed into vectors using the TOPO® TA Cloning® kit (Thermo Fisher) according to the manufacturer's protocol. Clones were checked using PCR with M13 primers and the purified PCR product was sent for Sanger sequencing to the company Microsynth AG. The sequencing results were analysed by BLASTN against the original target gene to check similarity and specificity of the primers. For standard preparation, a 10-fold dilution series was prepared from 10^10^ to 10^0^ copies per μl and checked for optimal efficiency.

### Quantitative reverse-transcription PCR

Each RT-qPCR reaction (20 µl) was performed in triplicate containing 10 µl SYBR Green Master Mix (2X stock, Bio-Rad), 0.25 µM of forward and reverse primers and 2 µl of cDNA template. Thermal cycling was performed in a CFX96 Touch^TM^ Real Time PCR Detection System (Bio-Rad) with initial denaturation at 95°C for 3 min followed by 40 cycles of 30 sec denaturation at 95°C, 30 sec annealing at 55°C and 30 sec elongation at 72°C. Optical detection was performed during the elongation steps and a melt curve was generated from 65°C to 95°C after cycling. Data was analyzed using the Bio-Rad CFX software (Bio-Rad). Relative-fold changes of target gene transcripts were calculated relative to the housekeeping gene DNA-directed RNA polymerase alpha subunit using the 2^-ΔCt^ calculation [[21]](https://paperpile.com/c/F1FXsp/MDjcJ).

### Catalyzed reporter deposition-fluorescence in situ hybridisation (CARD-FISH)

Sediment samples from Svalbard, Portugal and Sweden were fixed with 4% formaldehyde for 3 hrs on ice and stored in PBS:ethanol (1:1) at -20°C using standard procedures [[22]](https://paperpile.com/c/F1FXsp/QnI0F). Cells were extracted from fixed sediment samples that were stored in PBS:ethanol (1:1) for CARD-FISH by a Nycodenz density gradient centrifugation procedure. Briefly, PBS:ethanol samples (500 µl) were pelleted and washed 2 times in PBS to remove ethanol. Sediments were resuspended in 1.8 ml PBS, then sodium pyrophosphate (0.1% final) and tween 20 (0.5% final) were added, and samples were vortexed for 30 mins at medium speed (*c*. 4-5) on a Vortex Genie 2 vortexer (Scientific Industries), wth tubes sealed with parafilm and taped-down horizontally on the vortex adapter. Samples were then sonicated on ice at 50% power for 30 sec and setting ‘5’ (UW 2070 needle, Bandelin Electronics, Germany). Each cell suspension was then made-up to 4 ml with PBS in 13.2 ml Thinwall Polypropylene Tubes (Beckman Coulter), and then 2 ml of Nycodenz solution (80% w/v) (Alere Technologies) was carefully injected under the cell suspension with a needle and syringe. Samples were then centrifuged for 90 mins at 4 ℃ in a SW 41 Ti Swinging-Bucket Rotor (Beckman Coulter) at 14000 g, with no deceleration when stopping. Total supernatant was collected, and ethanol was added to make a 1:1 final solution, and stored directly at -20 ℃ (carry-over Nycodenz did not effect downstream procedures). For CARD-FISH, samples (c. 250-500 µl) were gently filtered onto 0.2 micron polycarbonate (GTTP) filters (Millipore), followed by washing through 5 ml of PBS. Filters were then dried at 46℃ for 5 mins prior to commencing CARD-FISH procedures.

Hybridisations were performed using the 5’-horseradish peroxidase-labeled (HRP) probe Acido-Sva-34-HRP (5’-GACTTATGTCATTGAGGACTCATGCGG-3’) and unlabelled helper probes (5’-GGATAGCCTCGGGAAACCGAGGGTAA-3’) and (5’-TGAGGGGAAAGGCGGGG-3’), or with HoAc1402-HRP (5’-CTTTCGTGATGTGACGGG-3’) with competitor compHoAc1402 (5’-CTTTCGTGACGTGACGGG-3’) [[23]](https://paperpile.com/c/F1FXsp/BDQeq).

We designed the horse-radish peroxidase (HRP) labeled probe Acido-Sva-34-HRP (5’-GACTTATGTCATTGAGGACTCATGCGG-3’) that targeted full-length acidobacteriotal 16S rRNA sequences that were 100% identical to the most abundant amplicon derived acidobacteriotal ASV 2257. The probe theoretical specificity was evaluated with the TestProbe function of the SILVA server. It perfectly matched 3 sequences from the Acidobacteriota sub-division 23 clade, and 15 sequences from the same genus when up to 3 mismatches were allowed. Only few (*n*=2) non-target hits became evident when up to 4 mismatches were allowed. Therefore we deemed having four theoretical mismatches to non-target sequences should provide high discriminatory power.

Hybridisations were performed overnight (c. 14-16 hrs) at 28°C with 50% formamide to achieve bright enough signals for imaging, using previously described protocols for Gram-negative bacteria [[22]](https://paperpile.com/c/F1FXsp/QnI0F) and Oregon-Green 488-labeled tyramides. The same cell morphologies (but with weak signals) were also visualised when performing hybridisations at 46°C for 3-5 hrs with 30% formamide (stringent conditions predicted by MathFISH [[24]](https://paperpile.com/c/F1FXsp/IGbSj). The same morphologies were also detected with weak signals using the HolAc1402-HRP probe targeting the phylum Aciddobacteriota [[23]](https://paperpile.com/c/F1FXsp/BDQeq). Cells were also stained with DAPI and visualised with an inverted Leica TCS SP8X CLSM using appropriate excitation/emission settings for DAPI and the Oregon-Green 488-labeled tyramides.

### Phylogenetic analyses

Using ARB [[25]](https://paperpile.com/c/F1FXsp/J1Ovf), 16S rRNA gene sequences were aligned with SINA [[26]](https://paperpile.com/c/F1FXsp/QEhYO) and inserted into the available full reference tree of SILVA database release 138 using parsimony option to find closest relatives to use for references. *De novo* trees using only near full-length reference sequences (>1200 bp) were constructed using fastDNAML, RaxML and phyML, with the bacterial variable position filter and default settings for all. A consensus tree was generated from these three trees, and then the query sequences were inserted using the parsimony option.

For phylogenetic analyses of *dsrB*-OTU sequences, Acidobacteriota *dsrB*-OTU sequences were retrieved that averaged >0.1% among all samples. To simplify the phylogenetic tree, these nucleotide sequences were further dereplicated using CD-HIT [[14]](https://paperpile.com/c/F1FXsp/v4kYI) at 90% identity. The amplicon- and metagenome-derived sequences were translated into amino acid sequences and were added to a previously described DsrAB reference alignment [[27, 28]](https://paperpile.com/c/F1FXsp/vvDP9+89AQz) using MAFFT [[29]](https://paperpile.com/c/F1FXsp/iwOeN) with the options: mafft --addfragments --6merpair --reorder --thread -6. DsrA/B sequences were added to the consensus DsrAB tree from Müller [[28]](https://paperpile.com/c/F1FXsp/89AQz) in ARB using the parsimony option. The DsrB from the MAG AM2 was too short to obtain a reliable placement, and was therefore excluded. We also tested evolutionary placement algorithm (EPA) of RAxML, however, the fine-scale tree branching of inserted sequences that were closely related was not consistent with sequence distances. The final tree was visualized in iTOL [[30]](https://paperpile.com/c/F1FXsp/TL4ZO).

### Analyses of publicly available 16S rRNA gene and dsrAB sequence datasets

To explore Acidobacteriota 16S rRNA gene sequences among publicly available sequence datasets from marine sediments, the amplicon-derived 16S rRNA sequence of ASV_2257 (listed below) that represented the most abundant *Ca*. Sulfomarinibacter ASV from Acidobacteriota from Svalbard, was used as query in: i) a search of Short Read Archieves (SRA) via the IMNGS server [[31]](https://paperpile.com/c/F1FXsp/S2FS), using 100 bp as ‘min size’ and 95% as ‘similarity threshold’ (March 2020); and ii) a search of the NCBI-nr nucleotide database to find datasets from clone library-based studies, using BLASTN and default parameters (December 2020). SRA datasets were also identified manually from literature searches, and downloaded. Relative abundances of *Ca*. Sulfomarinibacter ASV_2257 relatives (>95% identity) from the IMNGS SRA search results were calculated directly from the resulting output. To determine the relative abundances of *Ca*. Sulfomarinibacter- and *Ca.* Polarisedimenticola-related sequences among datasets from clone library-based studies, sequences from studies that provided hits of >98% identity to the ASV_2257 query sequence via BLASTN were downloaded. To streamline the analyses, all sequences from each individual study were then classified using the naïve Bayesian classification method (with 50% cutoff) as implemented in *mothur*, and as described in the main text. That is, for datasets from clone library-based studies, relative abundances were determined from all sequences provided by a study, including sequences from multiple depths or sites within the same study. Sequences downloaded from SRA datasets were also classified using the same naïve Bayesian classification method (with 50% cutoff). For IMNGS SRA and downloaded SRA derived results, the highest relative abundance of *Ca*. Sulfomarinibacter (SD-23)-related and *Ca.* Polarisedimenticola (SD-22)-related sequences from any of the samples within a study, were presented.

>ASV_2257

TACGGAGGGGGCAAGCGTTATTCGGATTTACTGGGCGTAAAGCGCACGTAGGTGGCATCGTAAGTCAAAGGTGAAAGCCCTCGGCTCAACCGAGGAACTGCCTTTGAAACTGCTTTGCTTGAGTCCGGGAGGGGGGAGCGGAATTCCCAGTGTAGCGGTGAAATGCGTAGATACTGGGAGGAACACCGGTGGCGAAGGCGGCTCCCTGGACCGGTACTGACACTGAGGTGCGAAAGCGTGGGTAGCAAACAGG

To explore Acidobacteriota *dsrA*/*B* sequences among publicly available sequence datasets from marine sediments, we performed a literature search for relevant studies/datasets, and also performed BLASTN analyses against the NCBI-nr database to find datasets containing Acidobacteriota *dsrA*/*B* and with sufficient metadata (e.g., co-ordinates). Classification of the DsrA/B sequences from each dataset was performed using a combined phylogenetic and naïve Bayesian classification approach as previously described [[18]](https://paperpile.com/c/F1FXsp/WRvtI). As described above for the 16S rRNA analyses, the relative abundances of *dsrA*/*B* were obtained from all sequences provided by a study, including if they contained sequences from multiple depths or sites within the same study. Further, relative abundances were also taken directly from several studies where Acidobacteriota *dsrA*/*B* (uncultured family-level lineage 9) were explicitly presented [[32–34]](https://paperpile.com/c/F1FXsp/EnUK+CDs1+xnjN).

# Supplementary Results

### Read mapping to Acidobacteriota MAGs

Mapping of metagenomic reads to the acidobacteriotal MAGs supported the general trends from 16S rRNA amplicon analyses (Supp. Table 2). No read pairs mapped to the AM4 MAG from the three metagenomes from the top 20 cm of Station J, Smeerenbergfjorden. A maximum of only 0.0003% of reads mapped when the identity threshold was reduced as low as 90% (to account for possible divergent populations among fjords). In contrast, the AM4 MAG recruited 0.21% of reads in Van Kuelenfjorden metagenome (AC, 18 cmbsf), which was more than ten times the maximum amount mapped to any of the *Ca*. Sulfomarinibacter MAGs in the same sample. At maximum, each of the *Ca*. Sulfomarinibacter MAGs had more than 0.9% of reads mapped from the 17 cmbsf metagenome from Station J of Smeerenbergfjorden, with the *Ca*. Sulfomarinibacter MAG AM3-C recruiting the most with 0.25% of reads.

### Marine Acidobacteriota may respire additional electron acceptors

The MAGs from both *Ca*. Sulfomarinibacter and *Ca.* Polarisedimenticola svalbardensis MAG AM4 encoded both *cbb*_3_- and *aa*_3_-type cytochrome complexes, which may enable reduction of oxygen with either high or low affinity, respectively [[35]](https://paperpile.com/c/F1FXsp/65AQr). These are typically used for oxygen respiration, but may also function for oxygen detoxification in some anaerobes [[36]](https://paperpile.com/c/F1FXsp/oAUm7).

The *Ca*. Sulfomarinibacter MAGs AM1-3 had genes for a predicted periplasmic type-II NosZ complex (Supp. Table 5 and Supp. Fig. 8), which may allow the reduction of nitrous oxide. Type-II NosZ are typically associated with organisms that prefer low oxygen, have enhanced N_2_O affinity, and organisms lacking enzymes for complete denitrification [[37]](https://paperpile.com/c/F1FXsp/hzvC). No enzymes for other steps of denitrification were encoded in *Ca*. Sulfomarinibacter MAGs, although cytochrome tetrathionate reductases (Otr) may also have nitrite reducing activity [[38, 39]](https://paperpile.com/c/F1FXsp/MyyuV+shPKe).

Reductive dehalogenase homologs and associated membrane anchor subunits were encoded adjacent to each other in several *Ca*. Sulfomarinibacter MAGs (Supp. Table 5 and Supp. Fig. 9). These may function for terminal respiratory reduction of halogenated organics, or removal of halogens from organics to enable further catabolism [[40]](https://paperpile.com/c/F1FXsp/QEH94).

The *Ca.* Polarisedimenticola svalbardensis MAG AM4 also encoded genes for periplasmic nitrate reductases (Nap), as well as associated membrane anchors and electron transfer subunits. These could enable respiration with nitrate (Supp. Table 5). They also did not appear to be capable of full denitrification.

A CISM enzyme which was phylogenetically affiliated with respiratory arsenate reductases was also identified in the *Ca.* Polarisedimenticola svalbardensis MAG AM4 (Supp. Fig. 6). Three other CISM subunit A enzymes were encoded among the MAGs, but were not phylogenetically affiliated with any characterised proteins (Supp. Fig. 6). Their functions therefore could not be predicted.

### Carbohydrate active enzymes in marine versus peatland Acidobacteriota

In contrast to the marine Acidobacteriota, peatland *dsr*-harbouring Acidobacteriota had up to 2.9% of their gene complements encoding glycoside hydrolases [[41]](https://paperpile.com/c/F1FXsp/tK2gF) (Supp. Table 7). Other Acidobacteriota from soil also typically have around 3% of their genes encoding glycoside hydrolases [[42]](https://paperpile.com/c/F1FXsp/e4a6v). The marine MAGs also encoded fewer glycoside hydrolases compared to terrestrial Acidobacteriota we compared, i.e., a maximum of 20 glycoside hydrolases were encoded in MAG AM3-B, versus 246 for Acidobacteriota MAG SbA6 (Supp. Table 7).

### Genome comparisons reveal distinct properties among novel genera and adaptations to marine environments

For reciprocal BLASTP analyses of protein sequences from *Ca*. Sulfomarinibacter and *Ca*. P. svalbardensis, we first dereplicated protein sequences from the *Ca*. Sulfomarinibacter MAGs to form a ‘genus-level protein complement’. The reciprocal BLASTP identified 4067 proteins unique to *Ca*. Sulfomarinibacter, and 1395 proteins unique to *Ca*. P. svalbardensis. The higher number from *Ca*. Sulfomarinibacter likely reflects gene diversity among the different species of the genus. Of these, most were hypothetical proteins (58 and 60%). Mapping the unique sets of proteins to the eggNOG database identified proteins belonging to 1183 and 619 ‘functional descriptors’ were unique to *Ca*. Sulfomarinibacter and *Ca*. P. svalbardensis, respectively (Supp. Table 8). We then examined unique eggNOG functional descriptors and RAST annotations that were present in multiple copies among each set of proteins, i.e., those >2 standard deviations above the mean for each (excluding hypotheticals or unclassified functions) (Supp. Table 8). These are described in the main text. Among proteins unique to either *Ca*. Sulfomarinibacter MAGs or the *Ca*. P. svalbardensis MAG, but that have broadly similar functions were: various transcriptional regulators, glycosyltranferases, *c*-type cytochromes, transporters, and trimethylamine methyltransferases (Supp. Table 8). All unique protein annotations and eggNOG functional descriptors are listed in Supp. Table 8.

To compare gene contents of marine and terrestrial *dsr*-harbouring Acidobacteriota, we performed ortholog-group (OG) analysis of protein sequences from our marine MAGs and acidobacteriotal MAGs from a peatland [[41]](https://paperpile.com/c/F1FXsp/tK2gF). Proteins unique to marine or terrestrial genomes were then classified into Clusters of Orthologous Groups (COG) categories. This revealed proteins involved in ion-transport/metabolism, cell wall biogenesis, signal transduction, and carbohydrate metabolism, were the most common unique proteins among the two sets of genomes (excluding proteins with ‘unknown functions’) (Supp. Fig. 12).

### Phylogenetic and meta-analyses of Acidobacteriota 16S rRNA genes

Phylogenetic analysis of all Acidobacteriota 16S rRNA gene ASVs (*n*=162) from Smeerenbergfjorden samples showed Thermoanaerobaculia were the most diverse, comprising 48.4% of all Acidobacteriota ASVs (Supp. Fig. 14). *Ca*. Polarisedimenticolia and sub-division 3 sequences made-up 20.2 and 8.1% of the remaining Acidobacteriota ASV diversity, respectively. Almost all closest relatives to our ASVs were derived from marine sediments (Supp. Fig. 14). A 16S rRNA gene was identified in one MAG identified from preliminary binning of metagenomic data and had 99% ANI to *Ca*. Sulfomarinibacter kjeldsenii MAG AM3-C. This 16S rRNA sequence affiliated with the class Thermoanaerobaculia (Supp. Fig. 14).

We also examined the taxonomies of Acidobacteriota 16S rRNA gene sequences from marine sediments within the SILVA database [[43]](https://paperpile.com/c/F1FXsp/dXaCW). From 11,108 Acidobacteriota sequences, 6.9% (*n*=771) were from marine sediments. They were mostly affiliated with class Thermoanaerobaculia (29.3%), with sequences from *Ca*. Sulfomarinibacteraceae (‘sub-group 23’) clade (8%) and sub-group 10 (Sva-0725) (20.8%) being common (Supp. Fig. 14). The class-level clades *Ca*. Polarisedimenticolia (sub-group 22 by SILVA) (20.1%), sub-group 21 (9.9%) and Vicinamibacteria (17.7%) were also abundant (Supp. Fig. 14). This indicated the MAGs analysed in this study represent two of the major Acidobacteriota lineages present in marine sediments, i.e., Thermoanaerobaculia and *Ca*. Polarisedimenticolia.

Among publicly available 16S rRNA gene datasets that we analysed, sub-group 10 (Sva-0725) was also the most commonly detected Acidobacteriota lineage other than *Ca*. Sulfomarinibacteraceae or *Ca*. Polarisedimenticolia, being present in 68% of the datasets and averaging 0.8% relative abundance. This group therefore should be targeted in future work for a better understanding of marine Acidobacteriota.

### Distribution of abundant Acidobacteriota dsrB OTU

The *dsrB-*OTU-17 (100% identical to *dsrB* of *Ca*. Sulfomarinibacter AM3-B MAG) reached 2.4% of *dsrB* amplicons in station GK at 9 cmbsf and and 4.3% in station J at 23 cmbsf (results not shown). It was the third most abundant *dsrB*-OTU in station GK at 9 cmbsf, and the sixth most abundant *dsrB*-OTU in station J at 23 cmbsf. Acidobacteriota *dsrB* transcripts made-up around 4% of *dsrB* transcripts on average among all sediments analysed (Supp. Fig. 16)

# Supplementary Discussion

### Potential oxygen reducing capacity may be used for oxygen defence in Ca. Sulfomarinibacter

The *Ca*. Sulfomarinibacter MAGs indicated the bacteria could respire oxygen using terminal *cbb*_3_- and *aa*_3_-type cytochromes. However, the results suggested largely anaerobic lifestyles for *Ca*. Sulfomarinibacter, i.e., oxygen-sensitive hydrogenases, heterodisulfide reductase complexes, low-redox c3-type cytochromes, rubrerythrins that are common among anaerobes for reactive oxygen defence, and expansion and activity (inferred from 16S rRNA expression) of populations in subsurface, anoxic sediments. In support of this, a recent stable isotope probing experiment showed that Acidobacteriota populations from estuarine sediments were mostly active under anoxic conditions in microcosms [[44]](https://paperpile.com/c/F1FXsp/7Z1zA). *Ca*. Sulfomarinibacter were also in very low relative abundances in permeable sediments of the North Sea versus higher relative abundances in impermeable sediments (Fig. 7D), indicating a preference for low oxygen conditions. We therefore speculate the oxygen-reducing cytochromes are used for detoxification of oxygen, similar to strictly anaerobic sulfate-reducers such as *Desulfovibrio vulgaris* [[36]](https://paperpile.com/c/F1FXsp/oAUm7). Supporting this, the only isolate of the Thermoanaerobaculia, i.e., *Thermoanaerobaculum aquaticum*, is strictly anaerobic [[45]](https://paperpile.com/c/F1FXsp/ps4eF), yet it also encoded *aa*_3_-type cytochromes. This may therefore point to detoxification functions for these cytochromes among the Thermoanaerobaculia lineage.

### Different Acidobacteriota abundances among fjords may reflect redox metabolisms

The two predominant Acidobacteriota groups in Svalbard sediments, i.e., the *Ca*. Sulfomarinibacter and *Ca*. Polarisedimenticola, had completely different abundances among the fjords that have different biogeochemical properties. These differences were supported by both 16S rRNA gene amplicon sequencing and mapping of metagenomic reads to MAGs. We hypothesize the different abundances reflect their different overall redox metabolisms. The *Ca*. Polarisedimenticolia were more abundant in Van Keulenfjorden, which has very high iron and manganese inputs [[46]](https://paperpile.com/c/F1FXsp/Y9JT6). Accordingly, the *Ca*. Polarisedimenticola svalbardensis MAG had more capabilities to utilize high-potential electron acceptors such as oxygen, nitrate and oxidized metals. In line with this, a previously described ‘Mor1 acidobacterium’, which is related to *Ca*. Polarisedimenticola svalbardensis MAG AM4, was enriched under aerobic conditions from seawater [[47]](https://paperpile.com/c/F1FXsp/Xuw7O). The *Ca*. Polarisedimenticolia lineage therefore seems capable of thriving in high redox conditions. In contrast, populations of the putative sulfate-reducing *Ca*. Sulfomarinibacter were in very low relative abundances in Van Keulenfjorden, and were likely suppressed by metal-reducers that typically outcompete sulfate-reducers when reactive metal-oxides are available [[1, 48]](https://paperpile.com/c/F1FXsp/PbAdO+QNdua). Accordingly, in Van Keulenfjorden, biogeochemical measurements showed that iron reduction was the predominant terminal reduction process and sulfate reduction rates are very low
(<12 nmol cm^-3^ d^-1^, in core) [[46, 49]](https://paperpile.com/c/F1FXsp/3rxF1+Y9JT6). In contrast, the *Ca*. Sulfomarinibacter appear adapted to low redox environments, and this may help explain why they are more abundant in the reduced (visibly black) subsurface sediments of Smeerenburgfjorden.

**References from Supplementary Figure captions**

**Supplementary Figure 4**. [[50]](https://paperpile.com/c/F1FXsp/pmHbx)

**Supplementary Figure 6**. [[51]](https://paperpile.com/c/F1FXsp/xLuKB)

**Supplementary Figure 7**. [[10]](https://paperpile.com/c/F1FXsp/QZfqg) [[7]](https://paperpile.com/c/F1FXsp/LFrUH)

**Supplementary Figure 9**. [[52]](https://paperpile.com/c/F1FXsp/svupZ)

**Supplementary Figure 11**. [[53]](https://paperpile.com/c/F1FXsp/8pxfs) [[54]](https://paperpile.com/c/F1FXsp/VFKKU)

**Supplementary Figure 12**. [[41]](https://paperpile.com/c/F1FXsp/tK2gF)

# References for supplementary information

1. [Buongiorno J, Herbert LC, Wehrmann LM, Michaud AB, Laufer K, Røy H, et al. Complex Microbial Communities Drive Iron and Sulfur Cycling in Arctic Fjord Sediments. *Appl Environ Microbiol*](http://paperpile.com/b/F1FXsp/QNdua)*.* [2019;**85**.](http://paperpile.com/b/F1FXsp/QNdua)

2. [Angel R, Claus P, Conrad R. Methanogenic archaea are globally ubiquitous in aerated soils and become active under wet anoxic conditions. *ISME J*](http://paperpile.com/b/F1FXsp/n37i2)*.* [2012;**6**:847–862.](http://paperpile.com/b/F1FXsp/n37i2)

3. [Schubert M, Lindgreen S, Orlando L. AdapterRemoval v2: rapid adapter trimming, identification, and read merging. *BMC Res Notes*](http://paperpile.com/b/F1FXsp/Itilp)*.* [2016;**9**:88.](http://paperpile.com/b/F1FXsp/Itilp)

4. [Bolger AM, Lohse M, Usadel B. Trimmomatic: a flexible trimmer for Illumina sequence data. *Bioinformatics*. 2014;**30**:2114–2120](http://paperpile.com/b/F1FXsp/oYwJ0).

5. [Bushnell B, Rood J, Singer E. BBMerge--accurate paired shotgun read merging via overlap. *PLoS One* 2017;**12**.](http://paperpile.com/b/F1FXsp/YBqht)

6. [Yu NY, Wagner JR, Laird MR, Melli G, Rey S, Lo R, et al. PSORTb 3.0: improved protein subcellular localization prediction with refined localization subcategories and predictive capabilities for all prokaryotes. *Bioinformatics*](http://paperpile.com/b/F1FXsp/5gFc7)*.* [2010;**26**:1608–1615.](http://paperpile.com/b/F1FXsp/5gFc7)

7. [Bagos PG, Nikolaou EP, Liakopoulos TD, Tsirigos KD. Combined prediction of Tat and Sec signal peptides with hidden Markov models. *Bioinformatics*](http://paperpile.com/b/F1FXsp/LFrUH)*.* [2010;**26**:2811–2817.](http://paperpile.com/b/F1FXsp/LFrUH)

8. [Savojardo C, Martelli PL, Fariselli P, Casadio R. DeepSig: deep learning improves signal peptide detection in proteins. *Bioinformatics*](http://paperpile.com/b/F1FXsp/Jg0zX)*.* [2018;**34**:1690–1696.](http://paperpile.com/b/F1FXsp/Jg0zX)

9. [Krogh A, Larsson B, von Heijne G, Sonnhammer EL. Predicting transmembrane protein topology with a hidden Markov model: application to complete genomes. *J Mol Biol*](http://paperpile.com/b/F1FXsp/MhfPX)*.* [2001;**305**:567–580.](http://paperpile.com/b/F1FXsp/MhfPX)

10. [Sullivan MJ, Petty NK, Beatson SA. Easyfig: a genome comparison visualizer. *Bioinformatics*](http://paperpile.com/b/F1FXsp/QZfqg)*.* [2011;**27**:1009–1010.](http://paperpile.com/b/F1FXsp/QZfqg)

11. [Garber AI, Nealson KH, Okamoto A, McAllister SM, Chan CS, Barco RA, et al. FeGenie: A Comprehensive Tool for the Identification of Iron Genes and Iron Gene Neighborhoods in Genome and Metagenome Assemblies. *Front Microbiol*](http://paperpile.com/b/F1FXsp/GQtF5)*.* [2020;**11**:37.](http://paperpile.com/b/F1FXsp/GQtF5)

12. [Tsirigos KD, Elofsson A, Bagos PG. PRED-TMBB2: improved topology prediction and detection of beta-barrel outer membrane proteins. *Bioinformatics*](http://paperpile.com/b/F1FXsp/3yy4X)*.* [2016;**32**:i665–i671.](http://paperpile.com/b/F1FXsp/3yy4X)

13. [Zhang H, Yohe T, Huang L, Entwistle S, Wu P, Yang Z, et al. dbCAN2: a meta server for automated carbohydrate-active enzyme annotation. *Nucleic Acids Res*](http://paperpile.com/b/F1FXsp/RrVaH)*.* [2018;**46**:W95–W101.](http://paperpile.com/b/F1FXsp/RrVaH)

14. [Huang Y, Niu B, Gao Y, Fu L, Li W. CD-HIT Suite: a web server for clustering and comparing biological sequences. *Bioinformatics*](http://paperpile.com/b/F1FXsp/v4kYI)*.* [2010;**26**:680–682.](http://paperpile.com/b/F1FXsp/v4kYI)

15. [Emms DM, Kelly S. OrthoFinder: phylogenetic orthology inference for comparative genomics. *Genome Biol*](http://paperpile.com/b/F1FXsp/03IV3)*.* [2019;**20**:238.](http://paperpile.com/b/F1FXsp/03IV3)

16. [Huerta-Cepas J, Forslund K, Coelho LP, Szklarczyk D, Jensen LJ, von Mering C, et al. Fast Genome-Wide Functional Annotation through Orthology Assignment by eggNOG-Mapper. *Mol Biol Evol*](http://paperpile.com/b/F1FXsp/hN0qe)*.* [2017;**34**:2115–2122.](http://paperpile.com/b/F1FXsp/hN0qe)

17. [Herbold CW, Pelikan C, Kuzyk O, Hausmann B, Angel R, Berry D, et al. A flexible and economical barcoding approach for highly multiplexed amplicon sequencing of diverse target genes. *Front Microbiol*. 2015;**6**](http://paperpile.com/b/F1FXsp/JMNIC).

18. [Pelikan C, Herbold CW, Hausmann B, Müller AL, Pester M, Loy A. Diversity analysis of sulfite- and sulfate-reducing microorganisms by multiplex *dsrA* and *dsrB* amplicon sequencing using new primers and mock community-optimized bioinformatics. *Environ Microbiol*](http://paperpile.com/b/F1FXsp/WRvtI)*.* [2016;**18**:2994–3009.](http://paperpile.com/b/F1FXsp/WRvtI)

19. [Untergasser A, Cutcutache I, Koressaar T, Ye J, Faircloth BC, Remm M, et al. Primer3—new capabilities and interfaces. *Nucleic Acids Res*. 2012;**40**:e115–e115](http://paperpile.com/b/F1FXsp/GssgT).

20. [Kumar S, Stecher G, Tamura K. MEGA7: Molecular Evolutionary Genetics Analysis Version 7.0 for Bigger Datasets. *Mol Biol Evol*](http://paperpile.com/b/F1FXsp/bw2bI)*.* [2016; **33**: 1870–1874.](http://paperpile.com/b/F1FXsp/bw2bI)

21. [Schmittgen TD, Livak KJ. Analyzing real-time PCR data by the comparative C(T) method. *Nat Protoc*](http://paperpile.com/b/F1FXsp/MDjcJ)*.* [2008;**3**:1101–1108.](http://paperpile.com/b/F1FXsp/MDjcJ)

22. [Wendeberg A. Fluorescence *in situ* hybridization for the identification of environmental microbes. *Cold Spring Harb Protoc*](http://paperpile.com/b/F1FXsp/QnI0F)*.* [2010;db.prot5366.](http://paperpile.com/b/F1FXsp/QnI0F)

23. [Juretschko S, Loy A, Lehner A, Wagner M. The microbial community composition of a nitrifying-denitrifying activated sludge from an industrial sewage treatment plant analyzed by the full-cycle rRNA approach. *Syst Appl Microbiol*](http://paperpile.com/b/F1FXsp/BDQeq)*.* [2002;**25**:84–99.](http://paperpile.com/b/F1FXsp/BDQeq)

24. [Yilmaz LS, Parnerkar S, Noguera DR. mathFISH, a web tool that uses thermodynamics-based mathematical models for in silico evaluation of oligonucleotide probes for fluorescence in situ hybridization. *Appl Environ Microbiol*](http://paperpile.com/b/F1FXsp/IGbSj)*.* [2011;**77**:1118–1122.](http://paperpile.com/b/F1FXsp/IGbSj)

25. [Ludwig W, Strunk O, Westram R, Richter L, Meier H, Yadhukumar, et al. ARB: a software environment for sequence data. *Nucleic Acids Res*](http://paperpile.com/b/F1FXsp/J1Ovf)*.* [2004;**32**:1363–1371.](http://paperpile.com/b/F1FXsp/J1Ovf)

26. [Pruesse E, Peplies J, Glöckner FO. SINA: accurate high-throughput multiple sequence alignment of ribosomal RNA genes. *Bioinformatics*](http://paperpile.com/b/F1FXsp/QEhYO)*.* [2012;**28**:1823–1829.](http://paperpile.com/b/F1FXsp/QEhYO)

27. [Lagkouvardos I, Fischer S, Kumar N, Clavel T. Rhea: a transparent and modular R pipeline for microbial profiling based on 16S rRNA gene amplicons. *PeerJ*. 2017;**5**:e2836](http://paperpile.com/b/F1FXsp/vvDP9).

28. [Müller AL, Kjeldsen KU, Rattei T, Pester M, Loy A. Phylogenetic and environmental diversity of DsrAB-type dissimilatory (bi)sulfite reductases. *ISME J*](http://paperpile.com/b/F1FXsp/89AQz)*.* [2015;**9**:1152–1165.](http://paperpile.com/b/F1FXsp/89AQz)

29. [Katoh K, Misawa K, Kuma K-I, Miyata T. MAFFT: a novel method for rapid multiple sequence alignment based on fast Fourier transform. *Nucleic Acids Res*](http://paperpile.com/b/F1FXsp/iwOeN)*.* [2002;**30**:3059–3066.](http://paperpile.com/b/F1FXsp/iwOeN)

30. [Letunic I, Bork P. Interactive tree of life (iTOL) v3: an online tool for the display and annotation of phylogenetic and other trees. *Nucleic Acids Res*](http://paperpile.com/b/F1FXsp/TL4ZO)*.* [2016;**44**:W242–5.](http://paperpile.com/b/F1FXsp/TL4ZO)

31. [Lagkouvardos I, Joseph D, Kapfhammer M, Giritli S, Horn M, Haller D, et al. IMNGS: A comprehensive open resource of processed 16S rRNA microbial profiles for ecology and diversity studies. *Sci Rep*](http://paperpile.com/b/F1FXsp/S2FS)*.* [2016;**6**:33721.](http://paperpile.com/b/F1FXsp/S2FS)

32. [Marshall IPG, Ren G, Jaussi M, Lomstein BA, Jørgensen BB, Røy H, et al. Environmental filtering determines family-level structure of sulfate-reducing microbial communities in subsurface marine sediments. *ISME J*](http://paperpile.com/b/F1FXsp/EnUK)*.* [2019;**13**:1920–1932.](http://paperpile.com/b/F1FXsp/EnUK)

33. [Pavloudi C, Oulas A, Vasileiadou K, Kotoulas G, De Troch M, Friedrich MW, et al. Diversity and abundance of sulfate-reducing microorganisms in a Mediterranean lagoonal complex (Amvrakikos Gulf, Ionian Sea) derived from *dsrB* gene. *Aquatic Microbial Ecology*. 2017;**79**:209–219](http://paperpile.com/b/F1FXsp/CDs1).

34. [Pelikan C, Jaussi M, Wasmund K, Seidenkrantz M-S, Pearce C, Kuzyk ZZA, et al. Glacial Runoff Promotes Deep Burial of Sulfur Cycling-Associated Microorganisms in Marine Sediments. *Front Microbiol*](http://paperpile.com/b/F1FXsp/xnjN)*.* [2019; **10**: 2558.](http://paperpile.com/b/F1FXsp/xnjN)

35. [Borisov VB, Gennis RB, Hemp J, Verkhovsky MI. The cytochrome *bd* respiratory oxygen reductases. *Biochim Biophys Acta*](http://paperpile.com/b/F1FXsp/65AQr)*.* [2011;**1807**:1398–1413.](http://paperpile.com/b/F1FXsp/65AQr)

36. [Lamrabet O, Pieulle L, Aubert C, Mouhamar F, Stocker P, Dolla A, et al. Oxygen reduction in the strict anaerobe *Desulfovibrio vulgaris* Hildenborough: characterization of two membrane-bound oxygen reductases. *Microbiology*](http://paperpile.com/b/F1FXsp/oAUm7)*.* [2011;**157**:2720–2732.](http://paperpile.com/b/F1FXsp/oAUm7)

37. [Bertagnolli AD, Konstantinidis KT, Stewart FJ. Non-denitrifier nitrous oxide reductases dominate marine biomes. *Environ Microbiol Rep*](http://paperpile.com/b/F1FXsp/hzvC)*.* [2020;**12**:681–692.](http://paperpile.com/b/F1FXsp/hzvC)

38. [Buckley A, MacGregor B, Teske A. Identification, Expression and Activity of Candidate Nitrite Reductases From Orange *Beggiatoaceae*, Guaymas Basin. *Front Microbiol*](http://paperpile.com/b/F1FXsp/MyyuV)*.* [2019;**10**: 644.](http://paperpile.com/b/F1FXsp/MyyuV)

39. [Atkinson SJ, Mowat CG, Reid GA, Chapman SK. An octaheme *c*-type cytochrome from *Shewanella oneidensis* can reduce nitrite and hydroxylamine. *FEBS Lett*](http://paperpile.com/b/F1FXsp/shPKe)*.* [2007;**581**:3805–3808.](http://paperpile.com/b/F1FXsp/shPKe)

40. [Temme HR, Carlson A, Novak PJ. Presence, Diversity, and Enrichment of Respiratory Reductive Dehalogenase and Non-respiratory Hydrolytic and Oxidative Dehalogenase Genes in Terrestrial Environments. *Front Microbiol*](http://paperpile.com/b/F1FXsp/QEH94)*.* [2019;**10**:1258.](http://paperpile.com/b/F1FXsp/QEH94)

41. [Hausmann B, Pelikan C, Herbold CW, Köstlbacher S, Albertsen M, Eichorst SA, et al. Peatland Acidobacteria with a dissimilatory sulfur metabolism. *ISME J*](http://paperpile.com/b/F1FXsp/tK2gF)*.* [2018;**12**:1729–1742.](http://paperpile.com/b/F1FXsp/tK2gF)

42. [Eichorst SA, Trojan D, Roux S, Herbold C, Rattei T, Woebken D. Genomic insights into the Acidobacteria reveal strategies for their success in terrestrial environments. *Environ Microbiol*](http://paperpile.com/b/F1FXsp/e4a6v)*.* [2018;**20**:1041–1063.](http://paperpile.com/b/F1FXsp/e4a6v)

43. [Quast C, Pruesse E, Yilmaz P, Gerken J, Schweer T, Yarza P, et al. The SILVA ribosomal RNA gene database project: improved data processing and web-based tools. *Nucleic Acids Res*](http://paperpile.com/b/F1FXsp/dXaCW)*.* [2013; **41**:D590–6.](http://paperpile.com/b/F1FXsp/dXaCW)

44. [Coskun ÖK, Özen V, Wankel SD, Orsi WD. Quantifying population-specific growth in benthic bacterial communities under low oxygen using H_2_^18^O. *ISME J*](http://paperpile.com/b/F1FXsp/7Z1zA)*.* [2019;**13**:1546–1559.](http://paperpile.com/b/F1FXsp/7Z1zA)

45. [Losey NA, Stevenson BS, Busse H-J, Sinninghe Damsté JS, Rijpstra WIC, Rudd S, et al. *Thermoanaerobaculum aquaticum* gen. nov., sp. nov., the first cultivated member of *Acidobacteria* subdivision 23, isolated from a hot spring. *Int J Syst Evol Microbiol*](http://paperpile.com/b/F1FXsp/ps4eF)*.* [2013;**63**:4149–4157.](http://paperpile.com/b/F1FXsp/ps4eF)

46. [Wehrmann LM, Formolo MJ, Owens JD, Raiswell R, Ferdelman TG, Riedinger N, et al. Iron and manganese speciation and cycling in glacially influenced high-latitude fjord sediments (West Spitsbergen, Svalbard): Evidence for a benthic recycling-transport mechanism. *Geochim Cosmochim Acta*](http://paperpile.com/b/F1FXsp/Y9JT6)*.* [2014;**141**:628–655.](http://paperpile.com/b/F1FXsp/Y9JT6)

47. [Cummings SL, Barbé D, Leao TF, Korobeynikov A, Engene N, Glukhov E, et al. A novel uncultured heterotrophic bacterial associate of the cyanobacterium *Moorea producens* JHB. *BMC Microbiol*](http://paperpile.com/b/F1FXsp/Xuw7O)*.* [2016;**16**:198.](http://paperpile.com/b/F1FXsp/Xuw7O)

48. [Froelich PN, Klinkhammer GP, Bender ML, Luedtke NA, Heath GR, Cullen D, et al. Early oxidation of organic matter in pelagic sediments of the eastern equatorial Atlantic: suboxic diagenesis. *Geochim Cosmochim Acta*](http://paperpile.com/b/F1FXsp/PbAdO)*.* [1979;**43**:1075–1090.](http://paperpile.com/b/F1FXsp/PbAdO)

49. [Michaud AB, Laufer K, Findlay A, Pellerin A, Antler G, Turchyn AV, et al. Glacial influence on the iron and sulfur cycles in Arctic fjord sediments (Svalbard). *Geochim Cosmochim Acta*](http://paperpile.com/b/F1FXsp/3rxF1)*.* [2020;**280**:423–440.](http://paperpile.com/b/F1FXsp/3rxF1)

50. [Kern M, Klotz MG, Simon J. The Wolinella succinogenes mcc gene cluster encodes an unconventional respiratory sulphite reduction system. *Mol Microbiol*](http://paperpile.com/b/F1FXsp/pmHbx)*.* [2011;**82**:1515–1530.](http://paperpile.com/b/F1FXsp/pmHbx)

51. [Duval S, Ducluzeau A-L, Nitschke W, Schoepp-Cothenet B. Enzyme phylogenies as markers for the oxidation state of the environment: the case of respiratory arsenate reductase and related enzymes. *BMC Evol Biol*](http://paperpile.com/b/F1FXsp/xLuKB)*.* [2008;**8**:206.](http://paperpile.com/b/F1FXsp/xLuKB)

52. [Hug LA, Maphosa F, Leys D, Löffler FE, Smidt H, Edwards EA, et al. Overview of organohalide-respiring bacteria and a proposal for a classification system for reductive dehalogenases. *Philos Trans R Soc Lond B Biol Sci*](http://paperpile.com/b/F1FXsp/svupZ)*.* [2013;**368**:20120322.](http://paperpile.com/b/F1FXsp/svupZ)

53. [Dyksma S, Pjevac P, Ovanesov K, Mussmann M. Evidence for H_2_ consumption by uncultured *Desulfobacterales* in coastal sediments. *Environ Microbiol*](http://paperpile.com/b/F1FXsp/8pxfs)*.* [2018;**20**:450–461.](http://paperpile.com/b/F1FXsp/8pxfs)

54. [Søndergaard D, Pedersen CNS, Greening C. HydDB: A web tool for hydrogenase classification and analysis. *Sci Rep*](http://paperpile.com/b/F1FXsp/VFKKU)*.* [2016;**6**:34212.](http://paperpile.com/b/F1FXsp/VFKKU)
